# Charge and Size Dual Switchable Nanocage for Novel Triple‐Interlocked Combination Therapy Pattern

**DOI:** 10.1002/advs.202000906

**Published:** 2020-08-04

**Authors:** Rui Yang, Zipeng Zhang, Shunli Fu, Teng Hou, Weiwei Mu, Shuang Liang, Tong Gao, Li Guan, Yuxiao Fang, Yongjun Liu, Na Zhang

**Affiliations:** ^1^ Department of Pharmaceutics Key Laboratory of Chemical Biology (Ministry of Education) School of Pharmaceutical Sciences Shandong University 44 Wenhuaxi Road Jinan Shandong 250012 China

**Keywords:** dual switchable nanocages, deep tumor penetration, pH‐triggered release, triple‐interlocked combination therapy, tumor‐associated macrophage repolarization

## Abstract

Combination therapy is a current hot topic in cancer treatment. Multiple synergistic effects elicited by combined drugs are essential in improving antitumor activity. Herein, a pH‐triggered charge and size dual switchable nanocage co‐loaded with abemaciclib and IMD‐0354 (PA/PI‐ND) is reported, exhibiting a novel triple‐interlocked combination of chemotherapy, immunotherapy, and chemoimmunotherapy. The charge reversal polymer NGR‐poly(ethylene glycol)‐poly(l‐lysine)‐dimethylmaleic anhydride (NGR‐PEG‐PLL‐DMA, ND) in PA/PI‐ND promotes the pH‐triggered charge reversal from negative to positive and size reduction from about 180 to 10 nm in an acidic tumor microenvironment, which greatly enhances cellular uptake and tumor tissue deep penetration. With the PA/PI‐ND triple‐interlocked combination therapy, the chemotherapeutic effect is enhanced by the action of abemaciclib to induce cell cycle arrest in the G1 phase, together with the reduction in cyclin D levels caused by IMD‐0354. The dual anti‐tumor promoting immunotherapy is achieved by abemaciclib selectively inhibiting the proliferation of regulatory T cells (Tregs) and by IMD‐0354 promoting tumor‐associated macrophage (TAM) repolarization from an M2 to M1 phenotype. Furthermore, PA/PI‐ND has improved anti‐tumor efficiency resulting from the third synergistic effect provided by chemoimmunotherapy. Taken together, PA/PI‐ND is a promising strategy to guide the design of future drug delivery carriers and cancer combination therapy.

## Graphical abstract

PA/PI‐ND can deliver to tumor tissues mediated by active ligand NGR. The interior PA and PI is released because of the switch of charge and size at the acidic tumor microenvironment to promote cellular uptake and deep penetration. PA/PI‐ND also pocesses a triple‐interlocked combination therapy pattern of chemotherapy, immunotherapy, and chemoimmunotherapy.

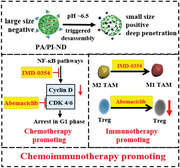

## 1 Introduction

Cancer is a malignant disease with a rapid‐growing mortality rate.^[^ ^1^ ^]^ Aberrant cell proliferation caused by uncontrolled cell division is one of the key features of cancer.^[^ ^2^ ^]^ Blocking cell division has been recognized as a potential therapeutic strategy.^[^ ^3^ ^]^ Cyclin‐dependent kinase 4 and 6 (CDK4/6) inhibitors have recently attracted much attention from researchers. CDK4/6 inhibitors can specifically target CDK4/6 and suppress retinoblastoma protein (RB) phosphorylation to arrest tumor cell cycle in G1 phase and inhibit cell proliferation.^[^ ^4^, ^5^ ^]^ Recent research has demonstrated that CDK4/6 inhibitor can selectively block the proliferation of immunosuppressive regulatory T cells (Tregs) to modulate the tumor immune‐microenvironment.^[^ ^6^, ^7^, ^8^ ^]^ Several CDK4/6 inhibitors have been recently approved for clinical therapy by the FDA, including abemaciclib, palbociclib, and ribociclib.^[^ ^9^ ^]^ In 2017, abemaciclib was approved as a monotherapy for hormone receptor (HR) positive, human epidermal growth factor receptor 2 (HER2) negative patients, with advanced or metastatic breast cancer. However, clinical data showed a median response duration of 8.6 months and an objective response rate of only 19.7%. The antitumor effect of abemaciclib still needs improvement through other strategies, such as combination therapy.^[^ ^10^ ^]^

The interaction between CDK4/6 and D‐type cyclin proteins (D1, D2, D3) catalyzes RB phosphorylation, promoting the cell cycle to enter the G1/S phase.^[^ ^11^ ^]^ Based on this pathway, our strategy is to develop a potential combination therapy pattern containing abemaciclib to improve the antitumor activity. Inhibiting expression of cyclin D is an effective method to enhance tumor cell cycle arrest with abemaciclib. Various cyclin D related signaling pathway inhibitors against estrogen, epidermal growth factor receptor (EGFR), phosphoinositide 3‐kinase (PI3K) and NF‐*κ*B hold great potential for this combination.^[^ ^4^, ^12^ ^]^ Cyclin D is one of the downstream proteins of NF‐*κ*B pathway.^[^ ^13^ ^]^ In this study, a NF‐*κ*B inhibitor (IMD‐0354) was selected to combine with abemaciclib to improve its therapeutic efficiency. IMD‐0354 could inhibit the NF‐*κ*B pathway, especially by restraining IKK*β*.^[^ ^14^ ^]^ In addition, IMD‐0354 exhibited significantly improved immunotherapeutic effects. Thus, combination of abemaciclib and the NF‐*κ*B inhibitor, IMD‐0354, could decrease the expression of the two major proteins and inhibit cell proliferation simultaneously.

On the other hand, combination of abemaciclib and IMD‐0354 was expected to promote immunotherapy synergistically. It has been demonstrated that a tumor immunosuppressive microenvironment caused by immunosuppressive cells limits the prognosis in cancer therapy. These cells included regulatory T cells (Tregs), regulatory B cells (Bregs), myeloid‐derived suppressor cells (MDSCs), M2 tumor‐associated macrophages (M2 TAMs), and so on.^[^ ^15^ ^]^ The proliferation of Tregs could be selectively inhibited by abemaciclib. We have previously demonstrated that IMD‐0354 can polarize M2 TAM to immune‐activating M1 TAM.^[^ ^16^ ^]^ Considering the effect on Tregs and M2 TAM, it was expected that the enhanced effect of reversing the immunosuppressive microenvironment could be achieved by the combination of abemaciclib and IMD‐0354.

Combining the enhanced chemo‐ and immunotherapeutic effect, the third synergistic effect (cancer chemoimmunotherapy) could be achieved by combination of abemaciclib and IMD‐0354. Chemoimmunotherapy has raised increasing interest for its enhanced antitumor effect from the synergism of chemo‐ and immune‐based therapeutic methods.^[^ ^17^ ^]^ These combination therapies have been practiced in preclinical studies and clinical trials.^[^ ^18^ ^]^ The combination of abemaciclib and IMD‐0354 possesses a variety of advantages, such as coordinating the triple therapeutic mechanisms, exerting complementary effective time, and overcoming chemotherapy resistance through immunotherapy.^[^ ^19^ ^]^ Taken above, it was aimed to promote the therapeutic efficiency of chemotherapy, immunotherapy, and chemoimmunotherapy, thus achieving a novel triple‐interlocked combination therapy pattern.

**Scheme 1:**
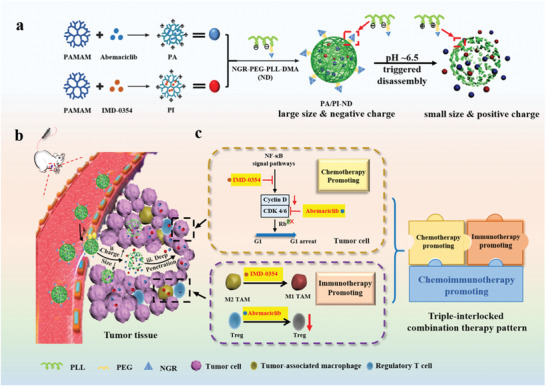
Schematic illustration of pH‐triggered dual charge and size responsive nanocage for novel triple‐interlocked combination therapy. a) Preparation of PA/PI‐ND; b) Delivery of PA/PI‐ND; i) PA/PI‐ND delivery to tumor tissues by the NGR ligand. ii) PA/PI‐ND gradually became positively charged and disintegrated, releasing PA and PI under conditions mimicking the acidic tumor microenvironment. iii) PA and PI reached the tumor interstitial region achieving deep tumor penetration. c) PA/PI‐ND exhibited a triple‐interlocked combination improving the therapeutic effect of chemotherapy, immunotherapy, and chemoimmunotherapy.

Drug‐delivery systems were essential to achieve synergistic efficiency from combination therapy. Ideal drug‐delivery systems could enhance tumor accumulation of drugs, facilitate cellular uptake and deep penetration into tumor tissues. Even though various co‐loaded drug carriers have been reported,^[^ ^20^ ^]^ there is a pressing need to develop drug carriers with all these functionalities, but with a simple preparation process.^[^ ^21^ ^]^ Herein, we propose a novel co‐delivery drug carrier, a dual size and charge switchable nanocage co‐loaded with abemaciclib and IMD‐0354 (PA/PI‐ND) (**Scheme** **1**). In the first step, the NGR peptide (sequence: GCNGRCGC) modified charge reversal polymer NGR‐poly(ethylene glycol)‐poly(l‐lysine)‐dimethylmaleic anhydride (NGR‐PEG‐PLL‐DMA, ND) was prepared. NGR peptide was selected as the targeting ligand as it is recognized as one of the specific CD13 ligands, which is overexpressed in tumor vascular epithelial cells.^[^ ^22^ ^]^ The small positive polyamidoamine (PAMAM) encapsulated abemaciclib (PA) and IMD‐0354 (PI) were prepared. PA and PI were cross‐linked with a pH‐responsive charge reversal polymer ND to form PA/PI‐ND. PA/PI‐ND was negatively charged at physiological conditions to prevent its clearance by the reticuloendothelial system (RES). After accumulation into tumor tissues mediated by NGR, the framework of PA/PI‐ND gradually decomposed in the acidic tumor microenvironment by charge conversion from negative to positive, triggering PA and PI to be released in the tumor microenvironment and then penetrate into deep tumor tissue. Furthermore, the antitumor activity was improved by the novel triple‐interlocked combination therapy of chemotherapy, immunotherapy, and chemoimmunotherapy.

In the present study, the nanocage PA/PI‐ND has been successfully prepared. The physicochemical properties were measured and the ability to switch charge and size was characterized. Tumor accumulation, co‐localization, and penetration of the nanocage were subsequently demonstrated in vitro and in vivo. To verify the enhanced efficiency of the triple‐interlocked combination therapy, the single synergistic effect provided by chemotherapy was evaluated using cytotoxicity assays and cycle arrest analyses on CT26 cells. The dual synergistic effect on reversing the tumor immunosuppressive microenvironment was investigated in the M2 TAM repolarization, and levels of cytokines, Tregs and T cells. The third enhancing effect exerted by chemoimmunotherapy was evaluated by antitumor activity in CT26 tumor‐bearing BALB/c mice. This research showed the potential of this novel triple‐interlocked combination therapy pattern in improving antitumor activity in vitro and in vivo.

## 2 Results and Discussion

### 2.1 Preparation and Characterization of Dimethylmaleic Anhydride‐Modified NGR‐Poly(ethylene glycol)‐Poly(l‐lysine) (NGR‐PEG‐PLL‐DMA, ND)

pH‐sensitive charge reversal materials were negative at physiological conditions (pH 7.4) to prevent clearance by the RES and could be switched into positively charged in acidic conditions for improving cellular uptake.^[^ ^23^ ^]^ Dimethylmaleic anhydride (DMA), tetrahydrophthalic anhydride (TDA) and *cis*‐aconitic acid anhydride (CA) modified poly(ethylene glycol)‐poly(l‐lysine) (MAL‐PEG‐PLL) was synthesized respectively (Figure S1, Supporting Information). The above materials all contained pH‐sensitive amide bonds which could be hydrolyzed in the tumor acidic microenvironment to achieve the charge switch. Neutrally charged succinic anhydride (SA)‐linked MAL‐PEG‐PLL was synthesized as control. The molecular structure of these materials was confirmed by proton nuclear magnetic resonance (^1^H‐NMR) analysis (Figures S2 and S3, Supporting Information). As shown in Figure S2 in the Supporting Information, the chemical shifts of MAL‐PEG‐PLL were identified as follows: PLL (—CH—, 4.17 ppm, —CH_2_—, 2.7–3.0 ppm, and —CH_2_—, 1.0–2.0 ppm) and PEG (—CH_2_—, 3.0–3.4 ppm), which proved the successful synthesis of MAL‐PEG‐PLL. As shown in Figure S4 in the Supporting Information, the peaks at 1650 and 1550 cm^−1^ correspond to the amide I and II bond, respectively. The peaks at 1240 and 1280 cm^−1^ could be attributed to the C—O—C stretching vibration in the PEG segment. The data also demonstrated that PEG was successfully modified to PLL. It was demonstrated by GPC (Figure S5 and Table S1, Supporting Information). The results of GPC showed that the molecular weight of MAL‐PEG‐PLL was increased than PLL, indicating the successful syntheses. The chemical shifts of 1.9 ppm (—CH_3_) were assigned to DMA, and the peaks at 2.1 ppm (—CH_2_—) and 1.5 ppm (—CH_2_—) were signals from TDA. The characteristic peaks at 2.3 and 5.9 ppm corresponded to signals from SA and CA, respectively (Figure S3, Supporting Information). As shown in FTIR, the peaks at 750–840 cm^−1^ was associated with the double bond. It was demonstrated that the successful syntheses of different charge reversal polymers.

**Figure 1:**
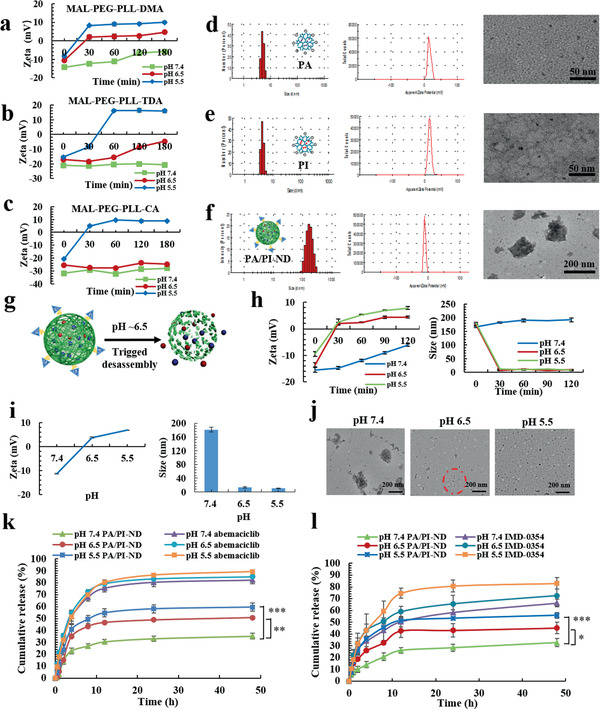
PA/PI‐ND exhibited charge and size switching capabilities. Charge responsiveness of a) MAL‐PEG‐PLL‐DMA, b) MAL‐PEG‐PLL‐TDA, and c) MAL‐PEG‐PLL‐CA incubated for different lengths of time and at different pH values (pH 7.4, 6.5, or 5.5). Size, zeta potential, and morphology of d) PA, e) PI, and f) PA/PI‐ND. g) Scheme of pH‐triggered disassembly of PA/PI‐ND. h) Zeta potential and size of PA/PI‐ND after incubation with PBS at different time‐periods and pH values. i) Zeta potential, size and j) morphology of PA/PI‐ND at different pH values after incubation for 1 h (Red circle represented disassembly of PA/PI‐ND). In vitro release behavior of k) abemaciclib and l) IMD‐0354 (*p < 0.05, **p < 0.01, ***p < 0.001, compared with PA/PI‐ND group under pH 7.4).

The above materials were optimized by comparing behaviors of charge switchable ability to select candidates for subsequent research. MAL‐PEG‐PLL‐DMA exhibited a suitable charge reversal ability under pH 6.5 and it could translate into 2 mV approximately within 30 min as measured by dynamic light scattering (DLS). The compounds MAL‐PEG‐PLL‐TDA and MAL‐PEG‐PLL‐CA were positively charged under pH 5.5 and negatively charged under pH 6.5 and 7.4. In comparison, MAL‐PEG‐PLL‐SA was negatively charged at pH 5.5, 6.5, and 7.4 (**Figure** **1**a–c; Figure S6, Supporting Information). Given the above results, MAL‐PEG‐PLL‐DMA was selected for this study as it exhibited a higher sensitivity of charge‐switchable ability under pH 6.5 compared to the other materials. The molecular structure of ND was confirmed by ^1^H‐NMR, GPC, and Energy Dispersive Spectrometer (EDS) (Figures S5 and S8 and Table S1, Supporting Information). The peaks at 1.3–2.0 ppm were attributed to NGR, and the peaks at 1.7–2.0 ppm corresponded to DMA. The molecular weight of ND was increased compared with it of MAL‐PEG‐PLL. Meanwhile, the EDS map showed that the elemental sulfur (S) was existed, indicating that NGR was modified to ND successfully. And as shown in Figure S8 in the Supporting Information, the concentration of sulfhydryl groups was not increased within 24 h, indicating that the disulfide bond of NGR was integrated during reaction.

### 2.2 Characterization and pH‐Triggered Release of PA/PI‐ND

Based on the optimized pH‐sensitive charge reversal polymer ND, we designed a co‐loaded abemaciclib and IMD‐0354 nanocage (PA/PI‐ND) with a pH‐triggered charge and size switch capability. Briefly, abemaciclib‐loaded PAMAM (PA) and IMD‐0354 loaded PAMAM (PI) were prepared, respectively. As measured by DLS and transmission electron microscopy (TEM), the hydrodynamic diameters and zeta potentials of PA were 4.59 ± 0.38 nm and 15.7 ± 0.81 mV, respectively, which were similar to those of PI (5.84 ± 1.28 nm and 15.2 ± 1.86 mV), and were elliptic (Figure 1d,e; Table S2, Supporting Information). Subsequently, PA/PI‐ND was self‐assembled by electrostatic interactions between positive PA, PI and negative polymer ND. PA/PI‐ND were spheres with hydrodynamic diameters of 177.7 ± 5.807 nm and zeta potentials of −11.2 ± 0.45 mV, suggesting that PA and PI have successfully reacted with ND (Figure 1f; Table S2, Supporting Information). The size of PA/PI‐ND was not changed significantly (*p* > 0.05), and the zeta potential was slightly increased within 4 h and kept stable, indicating that PA/PI‐ND could be stable in 20% plasma within 48 h (Figure S10, Supporting Information).

PA/PI‐ND was designed to disassemble and release internal PA and PI once exposed in the tumor acidic microenvironment (Figure 1g). The pH triggered charge and size switching characteristics of PA/PI‐ND were measured by DLS. Zeta potentials remained negative within 120 min under pH 7.4 but gradually changed from −13.6 to +2 mV within 30 min due to the acid‐catalyzed cleavage of DMA monomer on ND (Figure 1h). Additionally, the zeta potentials of PA/PI‐ND were reversed from ≈−11 to +7 mV, and the average size decreased from ≈180 to 10 nm from pH 7.4 to 6.5 (Figure 1i). The disassembly of PA/PI‐ND was also confirmed from TEM images at different pH 7.4, 6.5, or 5.5 (Figure 1j). These results demonstrated that PA/PI‐ND could disassemble under pH 6.5 to release PA and PI.

The pH‐responsive drug release of PA and PI from PA/PI‐ND was studied by incubating the nanocage in PBS at pH 5.5, 6.5, and 7.4. The accumulative release rate of IMD‐0354 and abemaciclib from PA/PI‐ND significantly increased to ≈45% and 50% at pH 6.5, and 32% and 35% at pH 7.4 after incubation for 48 h, respectively (Figure 1k,l). Considering the above results, at acidic conditions the surface charge of PA/PI‐ND was converted from negative to positive by the hydrolysis of the amide bonds in ND. The release of abemaciclib and IMD‐0354 was triggered by the disassembly of PA/PI‐ND in an acidic microenvironment.

### 2.3 Tumor Accumulation and Co‐Localization Efficiency of PA/PI‐ND

**Figure 2:**
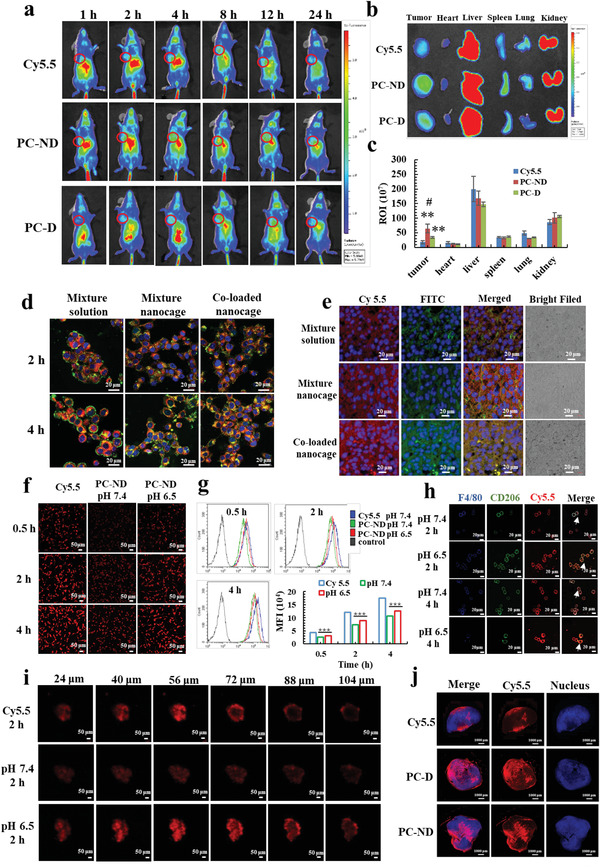
PA/PI‐ND exhibited characteristics of enhanced tumor accumulation and co‐localization efficiency, improved cellular uptake and penetration in an acidic microenvironment after drug delivery to tumor tissues. a) In vivo imaging of mice after administration with Cy5.5, PC‐D, and PC‐ND at different time intervals (tumors are marked with red circles). b) Ex vivo imaging and c) relative fluorescence intensity after mice were sacrificed after treatment 24 h (*n* = 3, **p < 0.01, compared with Cy5.5 group. #*p* < 0.05, compared with PC‐D group). d) Laser Confocal Microscopy (LSM) images of cellular uptake at different time intervals on CT26 cells (red, green and yellow colors represent Cy5.5, FITC and merged color, respectively, scale bar = 20 µm). e) LSM images of tumor tissue sections after 4 h administration (scale bar = 20 µm). f) LSM images and g) flow cytometric analysis of cellular uptake in CT26 cells at different pH values (***p < 0.001, scale bar = 50 µm). h) LSM images of cellular uptake of PC‐ND in RAW264.7 cells at different pH values (blue, green, and red colors represent F4/80, CD206, and Cy5.5, respectively, scale bar = 20 µm). i) In vitro penetration of Cy5.5 and PC‐ND in CT26 3D tumor spheroids after incubation for 2 h (scale bar = 50 µm). j) Full scan images of tumor tissue sections after administration of Cy5.5, PC‐D, and PC‐ND for 8 h (scale bar = 1000 µm).

The ability of PA/PI‐ND to target and accumulate in the tumor was demonstrated on CD13‐positive human umbilical vein endothelial cells (HUVEC) and BALB/c mice. Cy5.5‐loaded PAMAM (PC) was used to replace PA and PI to prepare PC‐ND. PC‐D (without NGR peptide) was designed as a control group. The red fluorescence intensity and cellular uptake rates of PC‐ND were significantly higher compared with PC‐D in HUVEC (Figure S11a–c, Supporting Information). To further demonstrate the tumor accumulation capability of PA/PI‐ND, real‐time imaging was applied. The PC‐ND group showed the highest fluorescence intensity at the tumor site (red circles) and the signals were 1.8‐ and 3.5‐fold higher than in the PC‐D group and cy5.5 group respectively, suggesting that PA/PI‐ND selectively targeted and accumulated in tumor sites (**Figure** **2**a–c). Quantification of the cy5.5 signal in other organs showed that the signal of free cy5.5 was mainly distributed in the liver area and was nearly absent in the tumor area. The distribution of PC‐ND and PC‐D was similar in other organs because of their similar physicochemical properties.

Subsequently, the co‐localization efficiency of PA/PI‐ND was evaluated by cellular uptake in CT26 cells. FITC‐labeled PAMAM (PF) and cy5.5‐labeled PAMAM (PC) replaced PA and PI, respectively. After incubating with CT26 cells for 2 and 4 h, the yellow fluorescence (caused by overlap of FITC and cy5.5) and co‐localization efficiency of the co‐loaded nanocage (PF/PC‐ND) were higher than that of a mixed fluorophore solution (FITC and Cy5.5) and of a mixed solution of nanocages (PF‐ND and PC‐ND) (Figure 2d; Figure S12a, Supporting Information). To further demonstrate the co‐localization efficiency of PA/PI‐ND, different samples were injected intravenously to BALB/c mice bearing CT26 cells; tumor sections were prepared for observation by LSM. The highest yellow fluorescence was observed in the co‐loaded nanocage group; green and red colors were produced in the solutions containing the mixed fluorophores and the mixed nanocages, respectively (Figure 2e). The quantification of the co‐localization efficiency was also carried out through ZEISS ZEN Lite Software. The co‐localization rate of co‐loaded nanocage was increased compared with mixture solution and mixture nanocage, indicating that the great co‐localization efficiency of co‐loaded nanocage. These results suggest that the PA/PI‐ND nanocage exhibited great co‐localization efficiency in tumor tissues (Figure S12, Supporting Ingormation).

### 2.4 Evaluation of Cellular Uptake and Deep Penetration into the Tumor

To demonstrate the enhanced cellular uptake and deep penetration in tumor tissue promoted by the charge and size dual switching properties, we first investigated the cellular uptake of PA/PI‐ND at pH 6.5 and 7.4. Fluorescent images and flow cytometric analysis showed that PC‐ND displayed a 1.6‐fold higher cellular uptake at pH 6.5 than at pH 7.4 in CT26 cells (Figure 2f,g) and in MCF‐7 cells (Figure S13, Supporting Information) after incubating for 4 h. Similar results were observed in RAW264.7 cells. The red fluorescence intensity in M2 macrophages was higher at pH 6.5 compared with pH 7.4 (Figure 2h). These results were confirmed by flow cytometry. As shown in Figure S14 in the Supporting Information, the cellular uptake of RAW 264.7 was significantly improved at pH 6.5 compared with pH 7.4 (*p* < 0.01). The enhanced cellular uptake could be attributed to the small size and positively charged PC released from PC‐ND in pH 6.5 that improved the interaction with the negatively charged cell membrane. In comparison, the PC‐ND nanocage was negatively charged and larger at pH 7.4.

Furthermore, the tumor penetration capacity of PA/PI‐ND was evaluated in CT26 3D tumor spheroids. Red fluorescence was observed in the tumor interior in the PC‐ND group under pH 6.5. In contrast, the fluorescence from Cy5.5 and PC‐ND (pH 7.4) was distributed around the edge of the 3D tumor spheroids (Figure 2i). Moreover, the tumor penetration capacity was investigated in vivo by administering different reagents for 8 h in BALB/c mice bearing CT26 cells. Most of the Cy5.5 fluorescence remained at the edge of the tumor, while the red fluorescence from PC‐D and PC‐ND were mostly located in the tumor interior (Figure 2j). Given these results, we concluded that the PA/PI‐ND nanocage exhibited deep tumor penetration capability made possible by the switch in charge and size in the acidic tumor microenvironment and the charge responsive disassembly of this material.

### 2.5 The Synergistic Chemotherapy Efficacy of PA/PI‐ND

**Figure 3:**
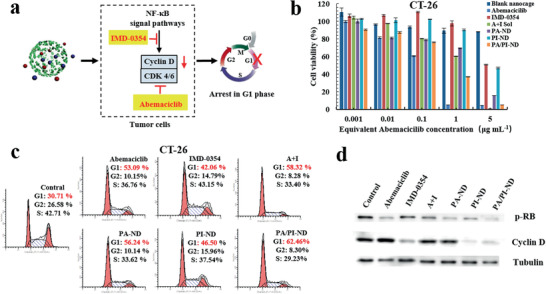
PA/PI‐ND exhibited a synergistic improvement on chemotherapy by combining the drug actions of abemaciclib and IMD‐0354. a) Scheme of one aspect that promoted the chemotherapeutic efficiency of PA/PI‐ND. b) Cell viability of PA/PI‐ND in CT26 cells in vitro. c) The effect of the induced cell cycle arrest after treatment with different samples for 24 h. d) Western blot of CT26 cells after treatment with different formulations for 24 h.

The combined therapy of abemaciclib and IMD‐0354 was expected to improve the chemotherapeutic efficiency. The synergistic effects between abemaciclib and IMD‐0354 were initially evaluated by using a MTT assay in CT‐26 and MCF‐7 cells, and the cooperativity index (CI) was calculated using the Chou‐Talalay method. The combination therapy had a high synergistic effect (CI was less than 1) when mass ratios of abemaciclib and IMD‐0354 were 5:0.5, 5:1, 5:2, and 5:3, indicating that the expected synergistic efficiency was obtained when the two drugs were internalized into tumor tissues (Figure S15a,b, Supporting Information). Next, the cytotoxicity was investigated in vitro after incubating different samples for 48 h on CT26 cells. An blank nanocage was used as control, which was similar in structure to PA/PI‐ND, except without any loaded drugs. No obvious toxicity was observed in the blank nanocage, demonstrating its biocompatibility and safety. The half‐maximal inhibitory concentration (IC_50_) of PA/PI‐ND was about 0.235 µg mL^−1^, showing a 1.8‐fold higher cytotoxicity than the abemaciclib solution (**Figure** **3**b; Table S3a, Supporting Information). In comparison, higher cytotoxicity was observed in the PA/PI‐ND group than in the PA‐ND and abemaciclib groups on MCF‐7 cells (Figure S16a and Table S3b, Supporting Information).

The combination therapy synergistic effect on cell cycle arrest was measured on CT26 and MCF‐7 cells after incubating different samples for 24 h. As illustrated in Figure 3c, the G1 phase proportion in the PA/PI‐ND group increased to 62.46% compared with PA‐ND (56.24%), PI‐ND (46.50%), and PBS (30.71%) groups. Similar results were observed on MCF‐7 cells (Figure S16b, Supporting Information). To study the mechanism of this synergistic effect, the levels of key proteins, including phosphorylated RB (p‐RB) and cyclin D, in CDK 4/6‐cyclin D pathways were measured through Western blot experiments. The p‐RB and cyclin D expression in PA/PI‐ND group decreased because abemaciclib could target CDK4/6 and inhibited RB phosphorylation while expressions of cyclin D could be inhibited by IMD‐0354 (Figure 3d). The above results ensured that PA/PI‐ND exhibited the expected enhanced performance on chemotherapy.

### 2.6 Characterization of Immunotherapy Promoting of PA/PI‐ND

**Figure 4:**
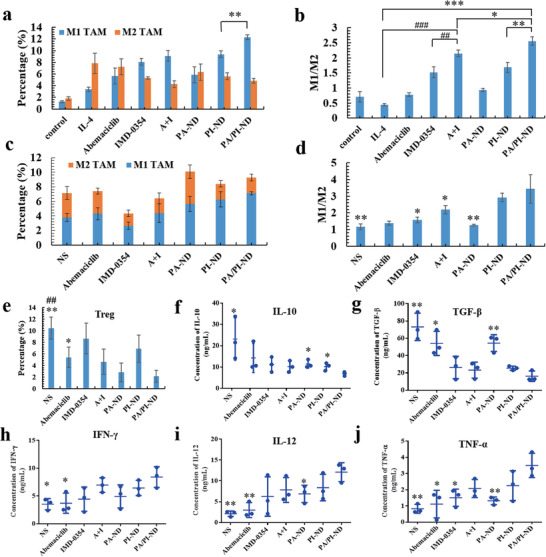
PA/PI‐ND improved immunotherapy efficiency and could reverse the immunosuppressive tumor microenvironment through the repolarization of M2 TAMs and decrease Treg cells. a) The number of M1 and M2 macrophages in RAW 264.7 cells (**p < 0.01). b) Ratio of M1 to M2 after treatment with different samples (*p < 0.05, **p < 0.01, ***p < 0.001, compared with PA/PI‐ND group; #*p* < 0.05, ###*p* < 0.001, compared with abemaciclib and IMD‐0354 mixture group). c) Numbers of M1 and M2 TAMs. d) Ratio of M1 to M2 TAMs in tumor tissues (*p < 0.05, **p < 0.01, compared with PA/PI‐ND group). e) Percentage of Treg cells in tumor tissues after treatment with different formulations. f–j) Cytokine levels in peripheral blood (*n* = 3, *p < 0.05, **p < 0.01, compared with PA/PI‐ND group).

Reprogramming the immunosuppressive tumor microenvironment improved antitumor activity. The second synergistic efficacy of the PA/PI‐ND nanocage on reversing immunosuppressive tumor microenvironment was evaluated. Repolarization of TAMs was evaluated on RAW264.7 cells after incubating with different samples for 24 h. As measured by flow cytometric analysis, in the PA/PI‐ND group the numbers of M2 macrophages (F4/80^+^CD206^+^) decreased and M1 macrophages (F4/80^+^CD86^+^) increased (**Figure** **4**a; Figure S17a, Supporting Information). The M1/M2 ratio in the PA/PI‐ND group was 1.5‐ and 5.8‐fold higher than the PI‐ND and IL‐4 groups, respectively (Figure 4b). Additionally, the in vivo M1/M2 TAM ratio in PA/PI‐ND group was 1.2‐, 2.2‐, 2.7‐, and 3.0‐fold higher in the PI‐ND, IMD‐0354, PA‐ND, and NS groups, respectively, suggesting that PA/PI‐ND could repolarize M2 TAMs to an M1 phenotype efficiently relieving the tumor immunosuppressive microenvironment (Figure 4c,d; Figure S17b, Supporting Information).

The enhanced immunotherapeutic effect was also assessed on the infiltration of immunogenic cells. The percentage of Tregs in the PA/PI‐ND group was lower than in the NS and in the abemaciclib groups, indicating that Tregs could be suppressed by PA/PI‐ND in the tumor microenvironment (Figure 4e). In addition, the PA/PI‐ND nanocage showed significantly higher infiltration of CD4^+^ and CD8^+^ T cells than the NS and the PA/ND groups in the tumor, resulting from the TAM repolarization by IMD‐0354 and improved CD4^+^ and CD8^+^ T cells proliferation by abemaciclib (Figure S18, Supporting Information).^[^ ^6^, ^22^, ^24^ ^]^ Furthermore, reversing the immunosuppressive microenvironment was also regulated by immunogenic cytokines, such as INF‐*γ*, IL‐12, TNF‐*α*, and immunosuppressive cytokines IL‐10 and TGF‐*β*.^[^ ^7^, ^25^ ^]^ The levels of IL‐10 and TGF‐*β* in the PA/PI‐ND group were lower than in the PA‐ND and in the NS groups. The cytokine levels of INF‐*γ*, IL‐12, and TNF‐*α* in the PA/PI‐ND group were higher than in the abemaciclib and NS groups (Figure 4f–j). The above results inferred that the immunosuppressive tumor microenvironment was relieved by the administration of the PA/PI‐ND nanocage.

### 2.7 Antitumor Efficacy of Synergistic Chemoimmunotherapy In Vivo

**Figure 5:**
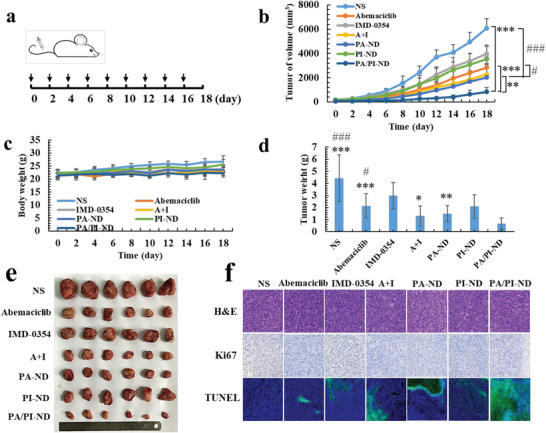
PA/PI‐ND enhanced the chemoimmunotherapeutic antitumor efficacy in CT26 tumor‐bearing BALB/c mice. a) Schedule of intravenous injection with different formulations in vivo (*n* = 6, black arrows represented administration time). b) Changes in tumor volume and c) body weight after intravenous injection with different formulations in vivo (**p < 0.01, ***p < 0.001, compared with PA/PI‐ND; #*p* < 0.05, ###*p* < 0.001, compared with PA‐ND, *n* = 6). d) Tumor weight at the endpoint (*p < 0.05, **p < 0.01, ***p < 0.001, compared with PA/PI‐ND; #*p* < 0.05, ###*p* < 0.001, compared with PA‐ND, *n* = 6). e) Microscopy images of ex vivo tumors after treatment with different samples. f) Immunohistochemical images of tumor tissue sections (H&E 400×, Ki67 200 ×, and TUNEL 20 × ).

Encouraged by the promoting chemo‐ and immuno‐ therapeutic tumor activity, the chemoimmunotherapy efficiency of PA/PI‐ND was evaluated on BALB/c mice bearing CT26 cells by intravenous injections of different formulations (NS, abemaciclib, IMD‐0354, abemaciclib and IMD mixture, PA‐ND, PI‐ND, and PA/PI‐ND). The administration schedule is shown in **Figure** **5**a, and the dose of abemaciclib and IMD‐0354 was 6 and 1.2 mg kg^−1^, respectively. Compared with the abemaciclib group, tumor volumes of PA‐ND and PA/PI‐ND group were significantly reduced (*p* < 0.05, *p* < 0.001), and the therapeutic effects of PA/PI‐ND were significantly improved compared with PA‐ND (*p *< 0.01). The tumor inhibition rate in PA/PI‐ND group was about 86.47% compared with the NS group (Figure 5b). The above results suggested that the antitumor activity efficiency could be improved when the mice were treated with the nanocage as it enhanced drug accumulation at tumor sites. At the same time, the combination of abemaciclib and IMD‐0354 could improve the anti‐tumor effect through the synergism of these two drugs. In addition, the mice body weights were measured and there was no significant decrease in body weight in each group. On day 18 after treatment with different samples, the mice were dissected to harvest the heart, liver, spleen, kidney, and lung. No obvious lesions in each group were observed as shown in the hematoxylin and eosin (H&E) stain sections, indicating a low systemic toxicity of PA/PI‐ND (Figure S19, Supporting Information). Hematological analyses of different formulations were carried out. As shown in Figure S20 in the Supporting Information, free abemaciclib, IMD‐0354 and a mixture of abemaciclib and IMD‐0354 exhibited certain toxicity compared with the NS groups, as reflected in the white blood cell (WBS) values which were outside the normal range and the increased levels of alanine aminotransferase (AST). After the mice were treated with PA/PI‐ND, there was no significant change in the hematological data compared with the NS group, indicating the low toxicity of PA/PI‐ND.

Tumor weights in the PA/PI‐ND group showed improved antitumor efficacy compared with the PA‐ND, abemaciclib and NS groups (Figure 5d,e). Cell proliferation and apoptosis in tumor tissues after treatment were measured by H&E, Ki67, and TUNEL staining. As shown in Figure 5f, large amounts of cell necrosis were observed in the PA/PI‐ND group. And Ki67 staining was used to observe proliferation of tumor cells. The positive regions (brown cells, Ki67 staining) represented the proliferation of tumor cells and consistent with the H&E staining results, cells cultured with PA/PI‐ND displayed less tumor cell growth. The TUNEL staining results (green fluorescence) showed a significant increase in apoptosis of tumor cells induced by PA/PI‐ND compared with other groups. Taken the above results, PA/PI‐ND has been demonstrated to exhibit superior antitumor activity in cancer chemoimmunotherapy and low systemic toxicity.

## 3 Conclusion

In summary, the co‐loaded abemaciclib and IMD‐0354 nanocage PA/PI‐ND was prepared with a pH‐triggered charge and size dual switchable capability which improved tumor accumulation, cellular uptake and deep penetration in the tumor acidic microenvironment. Furthermore, the PA/PI‐ND nanocage induced an effective cell cycle arrest, enhanced TAM repolarization, inhibited Treg cell function, and improved penetration of CD4^+^ and CD8^+^ T cells into the tumor tissue and antitumor activity utilizing the novel triple‐interlocked combination therapy on chemotherapy, immunotherapy, and chemoimmunotherapy. We suggest that the pH‐triggered dual charge and size switchable nanocage, PA/PI‐ND, efficiently enhanced the combination therapy and can potentially contribute to future developments of drug delivery carriers and cancer combination therapy.

## 4 Experimental Section

##### Materials

Abemaciclib was purchased from MedChemExpress Co. Ltd. IMD‐0354 was purchased from Selleckchem Co. Ltd. MAL‐PEG_2000_‐NHS was purchased from Beijing Kaizheng Biotech Co. Ltd (Beijing, China). Poly (L‐lysine) (PLL, *M*_w_ = 3–7 w), 2,3‐Dimethylmaleic Anhydride (DMA), *Cis*‐aconitic acid anhydride (CA), 3,4,5,6‐Tetrahydrophthalic anhydride (TDA) and succinic anhydride (SA) were purchased from Sigma‐Aldrich Co. Ltd (American). NGR peptide (sequence: GCNGRCGC) was obtained from Shanghai Apeptide Co. Ltd (Shanghai, China). Polyamindoamine (PAMAM‐G5, *M*_w_ = 28 826) was purchased from CY dendrimer technology Co. Ltd (Weihai, China). Cell Cycle and Apoptosis Analysis Kit was the product of Beyotime Biotechnology Co. Ltd (Shanghai, China). Alexa Fluor 647 anti‐mouse F4/80, Brilliant Violet 421 anti‐mouse CD206, PerCP/Cy5.5 anti‐mouse F4/80, Alexa Fluor 488 anti‐mouse CD86, APC anti‐mouse CD206, APC anti‐mouse CD3, FITC anti‐mouse CD4, PE anti‐mouse CD8, PE anti‐mouse CD25, and Alexa Fluor 488 anti‐mouse Foxp3 were purchased from Biolegend. ELISA kits were obtained from Dakewei Co. Ltd (Nanjing, China). All other reagents and solvents were obtained from Sinopharm Co., Ltd (Shanghai, China) and Sigma‐Aldrich Co., Ltd (Shanghai, China).

##### Cell Lines and Animals

Mouse colorectal cancer cells (CT26), human breast cancer cells (MCF‐7), human umbilical vein endothelial cells (HUVEC), and mouse macrophages (RAW 264.7) were obtained from Chinese Academy of Sciences (China). CT26 and HUVEC were incubated in RPMI‐1640, MCF‐7 cells were incubated in DMEM added 10% FBS, streptomycin and penicillin (1%). RAW 264.7 cell lines were cultured in DMEM added 10% FBS.

Female BALB/c mice (weight: 18–22 g) were purchased from SPF Beijing Biotechnology Co, Ltd. Mice were fed standardly food and permitted to drink freely. All experiments were implemented according to the Animal Management Rules of the Ministry of Health of the People's Republic of China and the Animal Experiment Ethics Review of Shandong University (Approval No. 18 014).

##### Synthesis of MAL‐PEG‐PLL and Various Charge Reversal Polymers

Briefly, PLL (50.0 mg) was dispersed ultrasonically in phosphate‐buffered saline (PBS, pH 9.0). Then, MAL‐PEG_2000_‐NHS (47.8 mg) was added dropwise to a PLL solution and the pH was adjusted to 7.4 using an aqueous 1.0 m NaOH solution. The reaction was stirred overnight under a N_2_ atmosphere. The reaction solution was then transferred to a dialysis tube (MWCO = 8–14 kD) against distilled water and then freeze‐dried to obtain PEG‐PLL. The structure of MAL‐PEG‐PLL was characterized by ^1^H NMR, Fourier‐transform infrared (FTIR) and gel permeation chromatography (GPC) using an aqueous mobile phase. DMA (three times the molar ratio of MAL‐PEG‐PLL) was then dissolved in dioxane and added dropwise to a MAL‐PEG‐PLL solution, stirring 4 h to obtain MAL‐PEG‐PLL‐DMA. The MAL‐PEG‐PLL‐TDA and MAL‐PEG‐PLL‐CA were prepared using the same procedure. As a pH‐insensitive control, SA modified MAL‐PEG‐PLL was synthesized as described above. The ^1^H NMR spectra of the final products were recorded on a 400 MHz spectrometer (in CD_4_O, 400 MHz) and they were also characterized by FTIR.

##### Evaluation of pH‐Responsive Charge Switchable Ability

To investigate the charge switching capacity of the MAL‐PEG‐PLL‐DMA, MAL‐PEG‐PLL‐TDA and MAL‐PEG‐PLL‐CA materials, the samples were dispersed into PBS (pH 5.5, 6.5, or 7.4) at 37 °C with gentle shaking. The solutions were then collected at pre‐designated time intervals and the zeta‐potentials were recorded. Similarly, the zeta‐potentials of MAL‐PEG‐PLL‐TDA and MAL‐PEG‐PLL‐CA were also measured. As a control, the zeta‐potential of the pH‐insensitive MAL‐PEG‐PLL‐SA material was measured at pH 5.5, 6.5, or 7.4, according to the same method.

##### Preparation of DMA Modified NGR‐PEG‐PLL‐DMA (ND)

The charge reversal polymer was prepared in two steps as shown in Figure S8 in the Supporting Information. First, NGR (5.0 mg) and MAL‐PEG‐ PLL (40.0 mg) were dissolved in 100 × 10^−3^ m phosphate buffer (pH 7.0 with 1 × 10^−3^ m EDTA). The reaction was stirred for 4 h at room temperature in a N_2_ atmosphere. The product was purified through dialysis (MWCO = 7 kD) then lyophilized. And in order to investigate the integrity of NGR, NGR and MAL‐PEG‐PLL were dissolved in 100 × 10^−3^ m phosphate buffer and then stirred for 4 h at room temperature in a N_2_ atmosphere. Then the reaction solution was taken out at different timed intervals (0, 0.5,1, 2, 4, 12, 24 h), and the concentration of sulfhydryl groups were determined by DTNB. DMA (three times the molar ratio of NGR‐PEG‐PLL) was then added dropwise to a solution of NGR‐PEG‐PLL and the pH value was maintained in the pH 9–10 range using 1.0 m NaOH. The reaction was stirred for an additional 6 h at room temperature. Subsequently, mixtures were purified by dialysis (MWCO = 3.5 kD) and then lyophilized. The final product was confirmed by ^1^H NMR (in CD_4_O, 400 MHz), FTIR, and GPC. The elemental analysis of ND was characterized by using an Energy Dispersive Spectrometer (EDS).

##### Preparation and Characterization of PA/PI‐ND

The assembly of PA/PI‐ND was driven by the electrostatic interactions between abemaciclib‐loaded PAMAM (PA), IMD‐0354‐loaded PAMAM (PI) and ND. First, PA and PI were prepared by the thin‐film hydration method. PAMAM and abemaciclib (molar ratio of PAMAM and abemaciclib was 10:1) were dissolved in methanol (1 mL) and the mixtures were stirred for 12 h using an eggplant‐shaped bottle. Methanol was then removed to form a uniform film layer by decompression with rotating. Next, distilled water was added and the film layer was hydrated at 50 ℃ to form PA. Meanwhile, the solution was filtered to remove free abemaciclib using a 0.22 µm syringe filter. PI was prepared following the same experimental procedure. Subsequently, PA and PI (mass ratio of abemaciclib and IMD‐0354 was 5:1) were added dropwise into a solution containing ND under vortex. Co‐loaded nanocage PA/PI‐ND was obtained by incubation for 30 min.

##### Characterizations of PA/PI‐ND

Particle size, size distribution and zeta potential of PA, PI, and PA/PI‐ND were measured by the dynamic light scattering (DLS) (NanoZS90, Malvern Instrument U.K). All measurements were repeated three times and results were described as mean ± SD (*n* = 3). The morphologies of PA, PI and PA/PI‐ND were examined using transmission electronic microscopy (TEM) (Hitachi, Japan). The samples were dripped into copper mesh, and then phosphotungstic acid was added to the copper mesh. Drug loading (DL) and encapsulation efficiency (EE) of abemaciclib and IMD‐0354 were measured at 296 and 267 nm respectively using HPLC (SPD‐10Avp Shimadzu pump, LC‐10Avp Shimadzu UV–vis Detector) after extraction with 90% methanol. DL and EE were measured using following formulations

(1) $$\mathrm{DL}\%=W_{\mathrm{drug}}/\left(W_{\mathrm{drug}}+W_{\mathrm{carrier}}\right)\times 100\%$$

(2) $$\mathrm{EE}\%=W_{\mathrm{drug}}/W_{\mathrm{total}\;\mathrm{drug}}\times 100\%$$

where *W*_drug_ and *W*_carrier_ epresent the weight of experimental drug and weight of carrier added to the system, respectively. *W*_total drug_ is the drug added to the system.

##### Stability Evaluation of PA/PI‐ND

PA/PI‐ND was diluted with 20% plasma and stored at 37 °C. The particle size and zeta potential of PA/PI‐ND was measured after incubation for 0, 1, 2, 4, 8, 12, 24, and 48 h, respectively.

##### In Vitro pH‐Sensitive Charge and Size Switchable Study of PA/PI‐ND

PA/PI‐ND was dispersed in PBS at pH 7.4, 6.5, or 5.5, respectively. PA/PI‐ND was incubated at 37 °C in a water bath. Then zeta potentials and sizes were monitored at different timed intervals (0, 30, 60, 90, 120 min). Additionally, PA/PI‐ND was added into PBS at pH 5.5, 6.5, or 7.4 and incubated for 1 h in thermostat water bath at 37 °C similarly. Zeta potentials and sizes were monitored by DLS. Each measurement was performed in triplicate. The morphologies of PA/PI‐ND in different pH values were observed through TEM after incubating for 1 h.

##### In Vitro Release Evaluation of PA/PI‐ND

Release profiles of abemaciclib and IMD‐0354 from PA/PI‐ND were determined by the dialysis method in vitro.^[^ ^26^ ^]^ Briefly, each sample (1 mL, abemaciclib, IMD‐0354 and PA/PI‐ND) was placed in dialysis bags (8–14 kD) and incubated in PBS (10 mL) buffer (pH 5.5, 6.5, or 7.4, with 1% Tween‐80) as release medium at 37 °C, respectively. All release mediums were withdrawn at the predesigned time intervals, and replaced with new release medium. Each sample was conducted in three times. Finally, the amounts of abemaciclib and IMD‐0354 released were determined by HPLC. The accumulative release percentage was measured as follow, and results were described as the mean ± standard deviation (SD)

(3) $$\mathrm{Cumulative}\;\mathrm{release}\left(\%\right)=Q_{n}/W\times 100\%=\sum_{i=0}^{n}C_{i}V/W\times 100\%$$

where *Q*_n_ represented the mass of accumulated released drug; *W* represented the mass of drug. *V* represented the release medium volume; *C*_i_ represented the concentration of drug in medium at each time intervals.

##### The Study of Active Targeting and Tumor Accumulation

To evaluate the active accumulation of PA/PI‐ND, its cellular uptake on HUVEC was measured and it's in vivo real‐time fluorescence imaging system (IVIS) spectrum (Caliper PerkinElmer, Waltham, MA, USA) was collected. Cy5.5 was loaded into PAMAM (PC) instead of abemaciclib and IMD‐0354 and incubated with ND to form Cy5.5‐labeled nanocage (PC‐ND). Similarly, PC‐D (without NGR) was prepared using the same method. HUVEC were seeded in a 12‐well plate at 1.5 × 10^5^ and incubated for ≈24 h. The media was replaced by fresh media containing PC‐D and PC‐ND (cy5.5 concentration of 2 µg mL^−1^) at pH 7.4 and incubated for 30 min, 2 and 4 h with 5% CO_2_ at 37 °C. The cells were then stained with Hoechst 33 342 and washed three times with PBS. Finally, the active targeting capability mediated by the NGR peptide on HUVEC was assessed by Laser Confocal Microscopy (LSM 780) (Carl Zeiss, Germany). To quantify the active targeting ability of the PC‐ND nanocage, HUVECs were treated with PC‐ND as described. Cells in each group were digested and collected, rinsed in PBS and the fluorescence intensities measured by CytoFLEX S flow cytometry (Beckman Coulter, USA).

The activing targeting and accumulation ability in tumor tissues were determined in vivo by IVIS spectrum. CT26 bearing BALB/c mice were selected as models of animal which were prepared though injection with 1 × 10^6^ CT26 cells into the right axilla intradermally. When tumor volumes reached about 200 mm^3^, mice were intravenous injections with 0.1 mL of cy5.5, PC‐D, and PC‐ND (the concentration of cy5.5 was 40 µg mL^−1^), respectively. After different time intervals (1, 2, 4, 8, 12, and 24 h) mice were put into the instrument after they were anesthetized. For ex vivo imaging, the tumor, heart, liver, spleen, lung, and kidney were obtained from mice after administration for 24 h, respectively. The real‐time images were observed by using IVIS spectrum. Results were processed by Living Image 3.1.

##### The Evaluation of Co‐Localization Efficiency In Vitro and In Vivo

To evaluate the co‐localization efficiency of PA/PI‐ND, abemaciclib and IMD‐0354 were replaced with Cy5.5 and FITC to assemble into Cy5.5‐ and FITC‐labeled co‐loaded nanocage (PC/PF‐ND). As controls, a Cy5.5‐labeled nanocage and a FITC‐labeled nanocage were prepared. CT26 cells were added to a 12‐well plate and incubated overnight. Different formulations (a mixed solution of Cy5.5 and FITC, a nanocage mixture and a co‐loaded nanocage) were added (the final concentration of Cy5.5 and FITC was 2 and 20 µg mL^−1^, respectively) and cultured for 2 and 4 h. The media was removed and the cells were stained with Hoechst 33 342 for 10 min. The CT‐26 cells were washed three times with PBS. The images were collected using LSM 780. To calculate the co‐localization efficiency, CT26 cells were treated as described above and the cells were digested and collected. The co‐localization efficiency was evaluated by CytoFLEX S. The evaluation of co‐localization efficiency was also measured on CT26 bearing BALB/c mice. Mice were administrated with cy5.5 and FITC mixture, mixture nanocage and co‐loaded nanocage when tumor volume reached 200 mm^3^. Mice were sacrificed after administrating different formulations for 4 h. Then tumors were harvested and fixed. Tumor sections were stained with DAPI and imaged by LSM 780. The images of tumor tissues’ sections were quantified through ZEISS ZEN Lite Software and the overlap coefficients of cy5.5 and FITC were calculated.

##### Cellular Uptake of PA/PI‐ND

Cy5.5 was selected as the fluorescent dye to prepare Cy5.5‐labeled nanocage PC‐ND. To evaluate cellular uptake ability on M2 macrophages, RAW 264.7 cells (5 × 10^4^) were seeded into laser confocal dishes and cultured for 12 h. IL‐4 (15 ng mL^−1^) was supplemented and then cultured for another 12 h to stimulate macrophages polarization into a M2 phenotype. The media was replaced with fresh media containing PC‐ND at pH 6.5 or 7.4 and incubated for 2 and 4 h at 37 °C with 5% CO_2_. The media was removed at the predesignated time. All macrophages were labeled with Alexa Fluor 647 anti‐mouse F4/80. M2 macrophages were simultaneously labeled with Alexa Fluor 647 anti‐mouse F4/80 and Brilliant Violet 421 anti‐mouse CD206. Finally, cellular uptake on M2 TAMs was imaged by using LSM 780 and fluorescence intensity of cellular uptake was quantified by CytoFLEX S.

##### Evaluation of PA/PI‐ND Tumor Penetration

Deep tumor penetration of PA/PI‐ND was characterized in vitro and in vivo. First, 3D tumor spheroids of CT26 cells were prepared. Briefly, agarose gel solutions (1.5%) were quickly added to a 96‐well plate, then a mixture of CT26 cells and matrigels (200 µL) were seeded at a density of 1 × 10^4^ cell per well. The cells were incubated for one week with 5% CO_2_ at 37 °C. The CT‐26 3D tumor spheroids were then removed and added into 12‐well plates. Cy 5.5, PC‐ND (pH 7.4) and PC‐ND (pH 6.5) were subsequently added. After incubation, CT‐26 3D tumor spheroids were rinsed in PBS. Penetration was observed using LSM 780 and the penetration capacity of PC‐ND was similarly examined in vivo. Different formulations (Cy5.5, PC‐D, and PC‐ND) were intravenously injected. After 8 h, tumors were harvested and made into frozen sections, then stained with DAPI and scanned.

##### Evaluation of the Synergistic Efficiency in Chemotherapy in Vitro

Abemaciclib and IMD‐0354 solutions were mixed at different mass ratios (abemaciclib/IMD‐0354 = 5:3, 5:2, 5:1, 10:1). Cells were added at a density of 5000 cells per mL in 96‐well plates and cultured for 12 h. Subsequently, cells were cultured for 48 h with different groups at gradually increasing abemaciclib concentrations (0.001, 0.01, 0.1, 1, and 5 µg mL^−1^). Then, 20 µL MTT solution was added to each well and cultured for another 4 h. The medium containing unreacted MTT was removed and DMSO was added to each well. Cell viability was measured at 570 nm by using a microplate reader (Synergy HTX Multi‐Mode Microplate Reader, BioTek, USA). The cytotoxicity of PA/PI‐ND was also determined using a MTT assay on CT26 and MCF‐7 cells using a similar procedure, except for the designed experimental groups. The samples were separated into different groups: 1) empty nanocage; 2) abemaciclib; 3) IMD‐0354; 4) abemaciclib and IMD‐0354 mixed solution (A+I); 5) PA‐ND; 6) PI‐ND; 7) PA/PI‐ND (abemaciclib concentration of 0.001, 0.01, 0.1, 1, and 5 µg mL^−1^, the concentration of IMD‐0354 was 1/5 of abemaciclib).

The induced cell cycle arrest was calculated on CT26 and MCF‐7 cells. Briefly, 5 × 10^5^ cells were seeded on 6‐well plates and cultured for 12 h. The media was replaced by different groups. After incubating for 24 h, cells were collected and fixed with 70% ethanol for 4 h at 4 ℃. Subsequently, different samples were stained with propidium iodide (PI) and measured by flow cytometry; data was analyzed using MELT32 software.

##### Western Blotting Experiment

After incubating with different groups for 24 h, CT26 cells were collected and denatured. Proteins were separated by SDS‐PAGE gel and transferred onto polyvinylidene difluoride membranes.^[^ ^27^ ^]^ After the blotted membranes were blocked in 5% skim milk for 30 min, the membranes were incubated with various primary antibodies overnight at 4 °C. Horseradish peroxidase–linked IgG secondary antibodies were incubated for another 4 h at room temperature and measured using chemiluminescence.

##### Evaluation of Reversing Immunosuppressive Tumor Microenvironment by PA/PI‐ND

Tumor‐associated macrophage (TAM) repolarization capacity was measured by flow cytometry respectively in vitro and in vivo as previous reported.^[^ ^28^ ^]^ 6 × 10^6^ RAW264.7 cells were incubated on 12‐well plates and cultured overnight. Then IL‐4 (15 ng mL^−1^(was supplemented to stimulate macrophages polarized into M2 type. After incubation for 12 h, media were replaced with fresh media containing different formulations (PBS, abemaciclib, IMD‐0354, abemaciclib and IMD‐0354 mixture, PA‐ND, PI‐ND, and PA/PI‐ND). After incubating for 24 h, all macrophages (F4/80^+^), M2‐type macrophages (F4/80^+^ CD206^+^) and M1‐type macrophages (F4/80^+^ CD86^+^) were marked with different immunofluorescent antibody. Next, the hypotypes of RAW264.7 were analyzed using flow cytometry. To determine repolarization ability in vivo, tumor tissues were collected, lapped and filtered using a copper network. The solutions of filtered tumor tissues were centrifuged at 1500 rpm for 10 min to obtained cells. Subsequently cells were marked antibodies as used in vitro. Then the cells were analyzed using flow cytometry.

The changes of Treg and T cells were also measured by flow cytometry. Briefly, tumor tissues were collected and filtered using a copper network. Lymphocytes were obtained by using 40% Percoll solution. Lymphocytes were marked with different anti‐mouse immunofluorescent antibody (FITC marked CD4, PE marked CD25, Alexa Fluor 488 marked Foxp3). Then the amounts of Treg were measured through flow cytometry. The cells with CD4^+^CD25^+^Foxp3^+^ were recognized to be Tregs. Lymphocytes were also marked with different anti‐mouse immunofluorescent antibody (APC marked CD3, FITC marked CD4, PE marked CD8). The cells with CD3^+^CD4^+^ were recognized to be CD4^+^ T cells, the cells with CD3^+^CD8^+^ were recognized to be CD8^+^ T cells.

##### Enzyme Linked Immunosorbent Assay

For ELISA assay, at 18th of the treatment, orbital bloods were taken from different groups of mice. IFN‐*γ*, IL‐12, TNF‐*α*, IL‐10, and TGF‐*β* was measured by ELISA kits (Dakewei, Nanjing, China) according to the operating instructions, respectively. Each sample was conducted in triplicate.

##### In Vivo Antitumor Efficacy

CT26 bearing BALB/c mice were selected as the animal models. CT26 cells (1 × 10^6^) in PBS (0.1 mL) were injected at right axillary subcutaneously. After few days, mice were divided into seven groups randomly (*n* = 6) when the volume of tumor was ≈100 mm^3^. The dose of abemacicilib and IMD‐0354 was 6.0 and 1.2 mg kg^−1^, respectively. Then mice were intravenously injected different formulations every 2 days as described in the following: 1) normal saline (NS); 2) abemaciclib; 3) IMD‐0354; 4) abemaciclib and IMD‐0354 mixture (A+I); 5) PA‐ND; 6) PI‐ND; 7) PA/PI‐ND. Within 18 days of first administration, tumor volume and body weight were recorded every two days. The volume of tumor was calculated according to this equation: (length × width^2^)/2, that the longest diameter was described as length and the widest diameter was described as width, respectively. On day 18, mice were sacrificed and the tumor was isolated in each group. Then the tumors were weighed and photographed.

##### Histological Evaluation

Tumor tissues were fixed in 4% formaldehyde and embedded in paraffin. Tumor sections were stained through H&E (hematoxylin and eosin), Ki67, and TUNEL, respectively. Finally, histological changes, apoptosis and proliferation of tumor tissues were observed.

##### Hematological Analysis

For hematological analysis, at 18th of the treatment, orbital bloods were taken from different groups of mice for serum biochemistry experiments and complete blood panel analysis.

##### Statistical Analysis

The statistical differences between different groups were calculated by the Student's t‐test, one‐way or two‐way ANOVA. *p* < 0.05 were considered statistically significant. All values are expressed as means ± SD.

## Conflict of Interest

The authors declare no conflict of interest.

## Supporting information

Supporting Information
