# Supplementary material for: Charge and Size Dual Switchable Nanocage for Novel Triple‐Interlocked Combination Therapy Pattern
Source: Adv Sci (Weinh). 2020 Aug 4;7(18):2000906. doi: 10.1002/advs.202000906 (PMC7509747; doi:10.1002/advs.202000906)
Supplement: Supplementary file 1 — Supporting Information [file ADVS-7-2000906-s001.pdf]

2020 Wiley-VCH GmbH

ADVANCED SCIENCE
Open Access

Supporting Information

for *Adv. Sci.,* DOI: 10.1002/advs.202000906

Charge and Size Dual Switchable Nanocage for Novel Triple-Interlocked Combination Therapy Pattern

Rui Yang, Zipeng Zhang, Shunli Fu, Teng Hou, Weiwei Mu, Shuang Liang, Tong Gao, Li Guan, Yuxiao Fang, Yongjun Liu, and Na Zhang**

Copyright WILEY-VCH Verlag GmbH & Co. KGaA, 69469 Weinheim, Germany, 2016.

Supporting Information

Charge and Size Dual Switchable Multistage Nanocage for Novel Triple-Interlocked Combination Therapy Pattern

*Rui Yang, Zipeng Zhang, Shunli Fu, Teng Hou, Weiwei Mu, Shuang Liang, Tong Gao, Li Guan, Yuxiao Fang, Yongjun Liu, * and Na Zhang**

Supplementary Figures and Tables

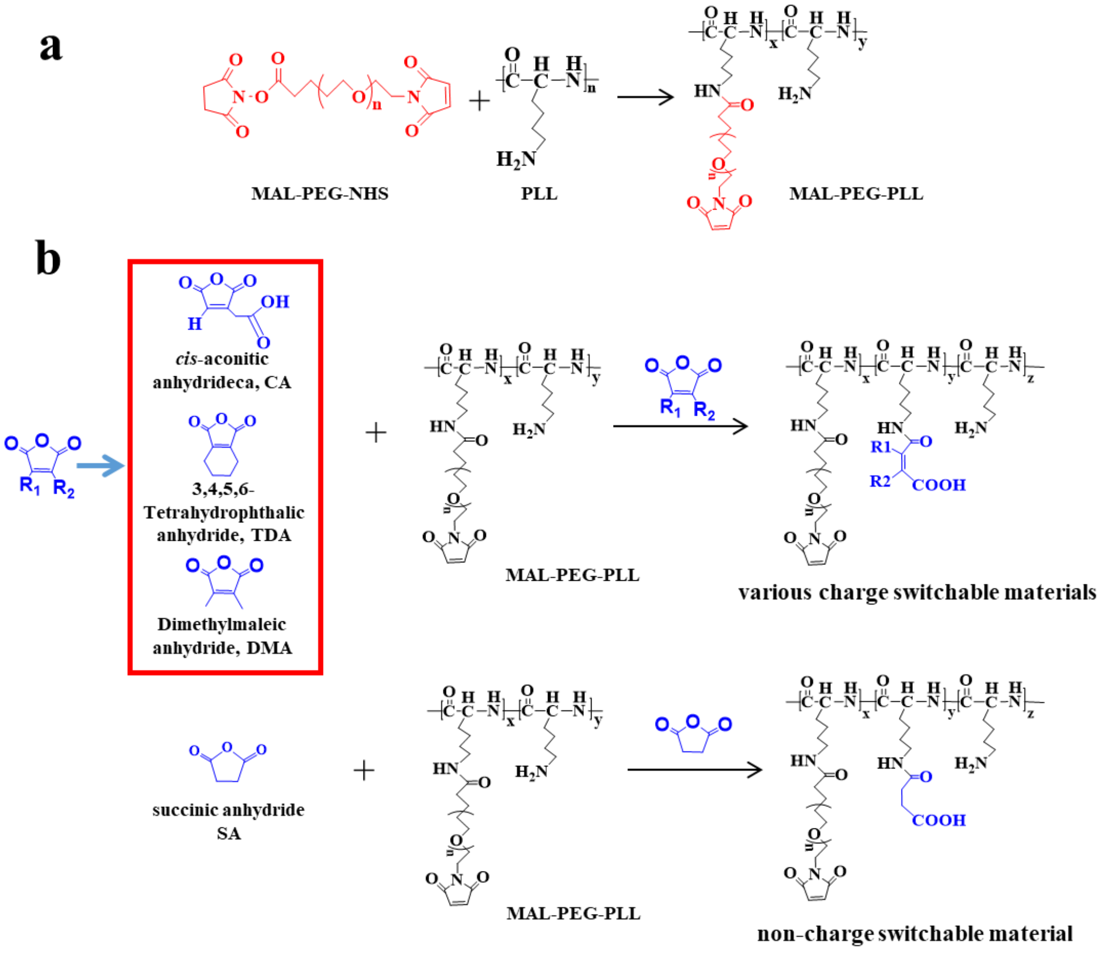

Figure S1. Synthetic routes of (a) MAL-PEG-PLL and (b) various charge switchable materials and non-charge switchable material.

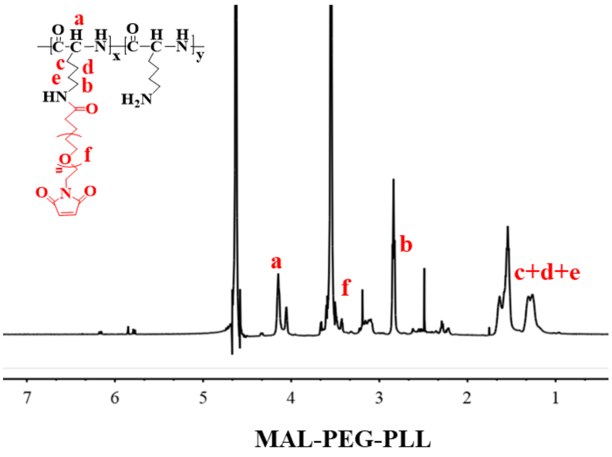

Figure S2. ^{1}H NMR spectra of MAL-PEG-PLL.

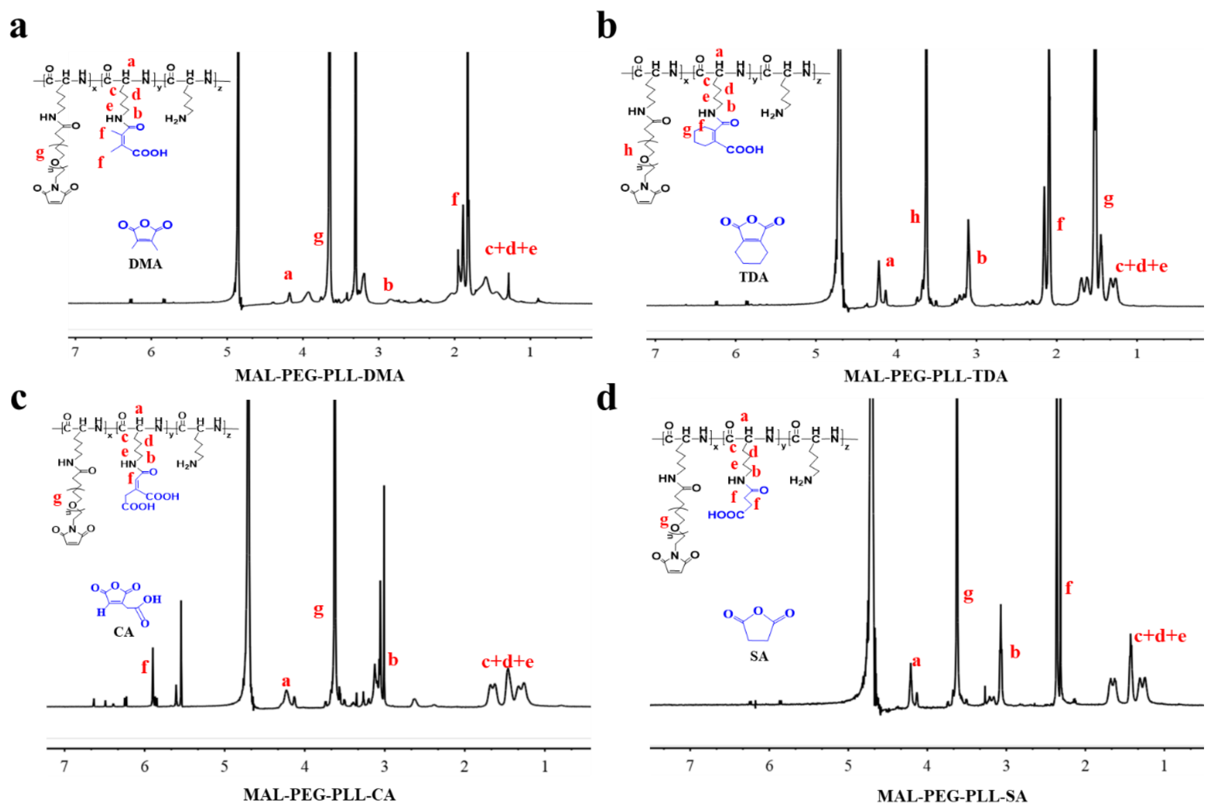

Figure S3. ^{1}H NMR spectra of (a-d) various pH sensitive charge reversal materials: (a) MAL-PEG-PLL-DMA; (b) MAL-PEG-PLL-TDA; (c) MAL-PEG-PLL-CA and (d) pH-insensitive material MAL-PEG-PLL-SA.

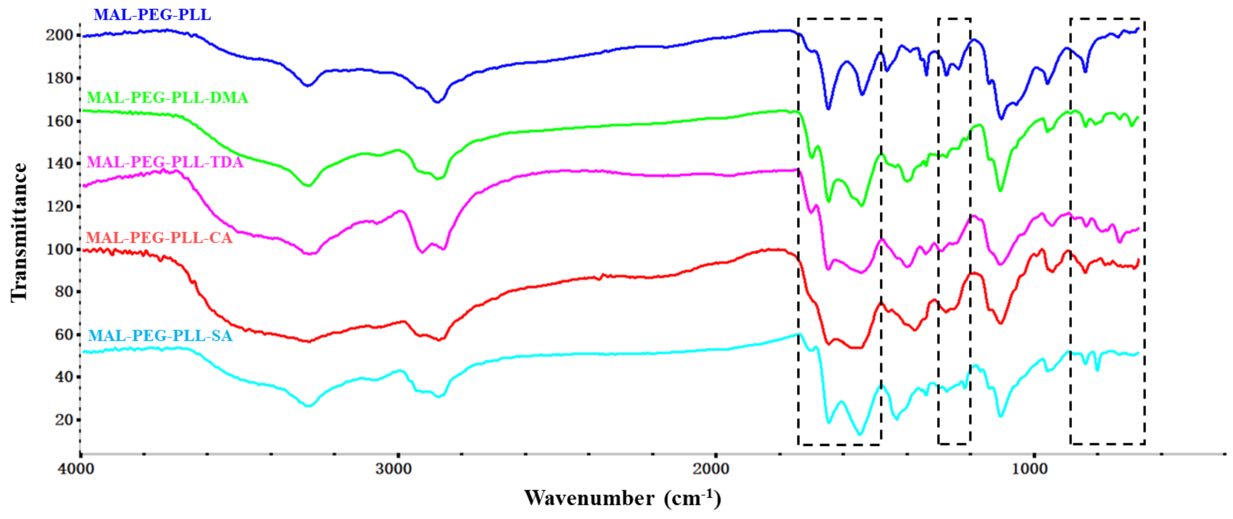

Figure S4. FTIR spectra of MAL-PEG-PLL and various pH sensitive charge reversal materials: MAL-PEG-PLL-DMA; MAL-PEG-PLL-TDA; MAL-PEG-PLL-CA and pH-insensitive material MAL-PEG-PLL-SA.

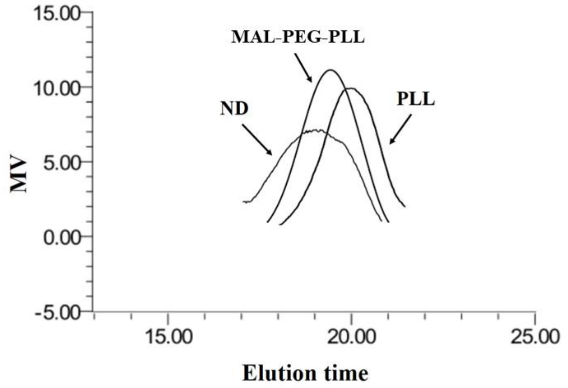

Figure S5. The GPC profiles of PLL, MAL-PEG-PLL and NGR-PEG-PLL-DMA (ND).

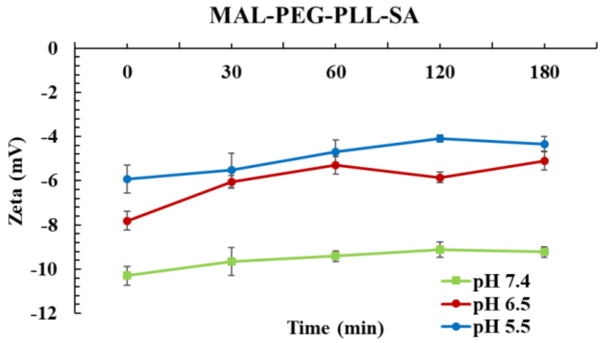

Figure S6. Charge switchable ability of MAL-PEG-PLL-SA incubated for different time at different pH values (pH 7.4, 6.5 or 5.5).

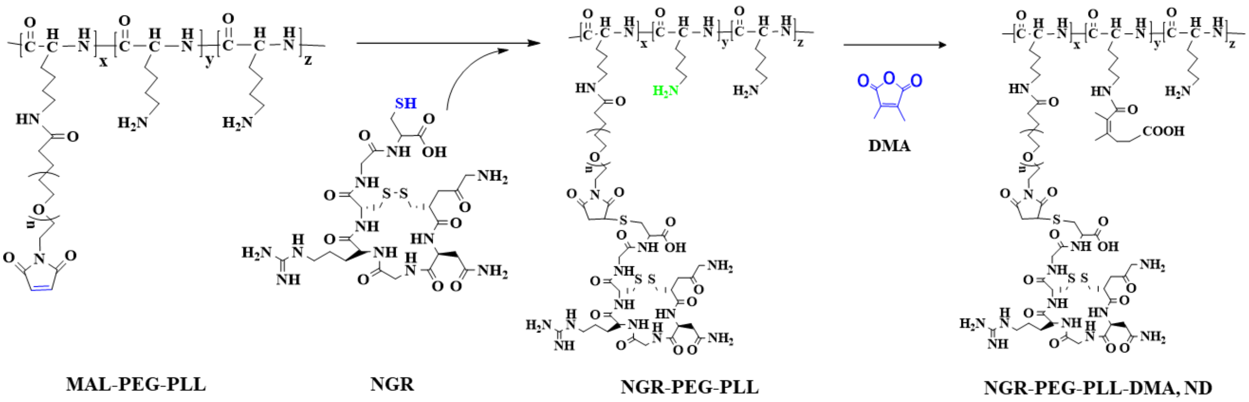

Figure S7. Synthetic routes of NGR-PEG-PLL-DMA (ND).

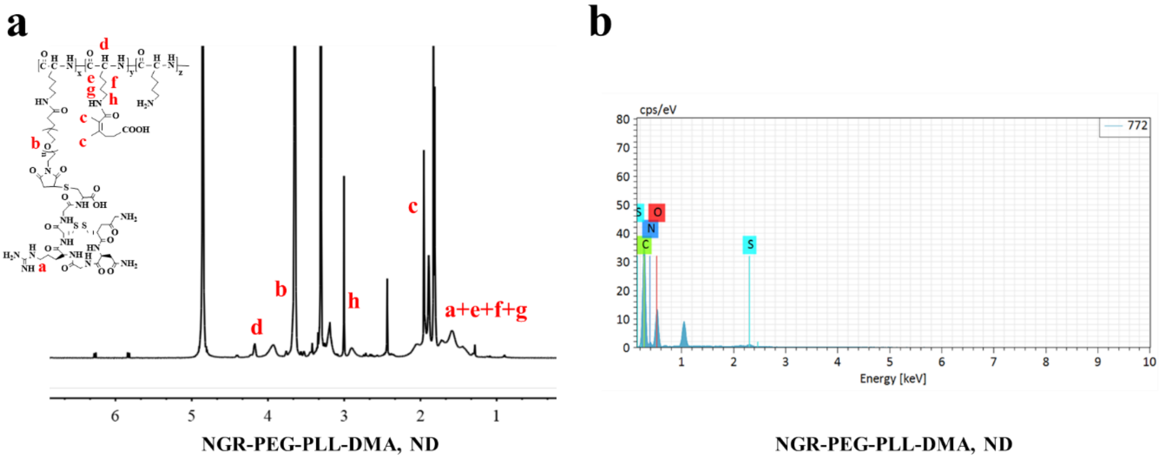

Figure S8. (a) ^{1}H NMR spectrum and (b) EDS of NGR-modified pH triggered charge switchable polymer NGR-PEG-PLL-DMA (ND).

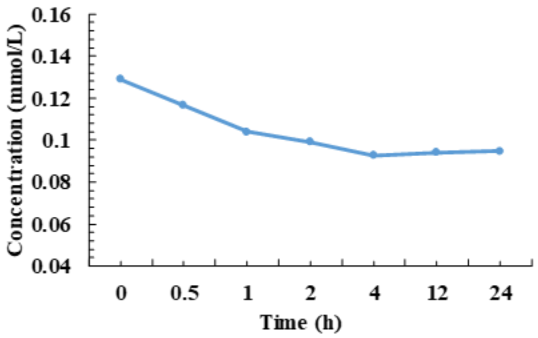

Figure S9. The changes of concentration of sulfydryl during reaction by DTNB assay.

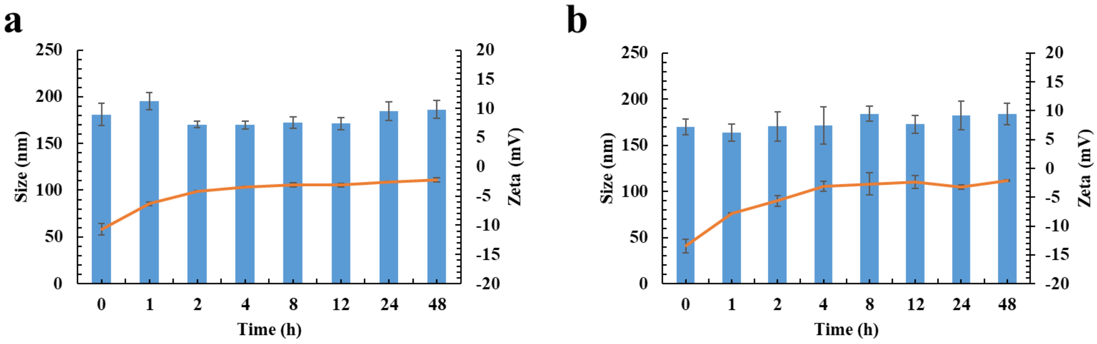

Figure S10. The stability of PA/PI-ND. Size and zeta potential changes of PA/PI-ND at (a) pH 7.4 and (b) 20% plasma for 48 h

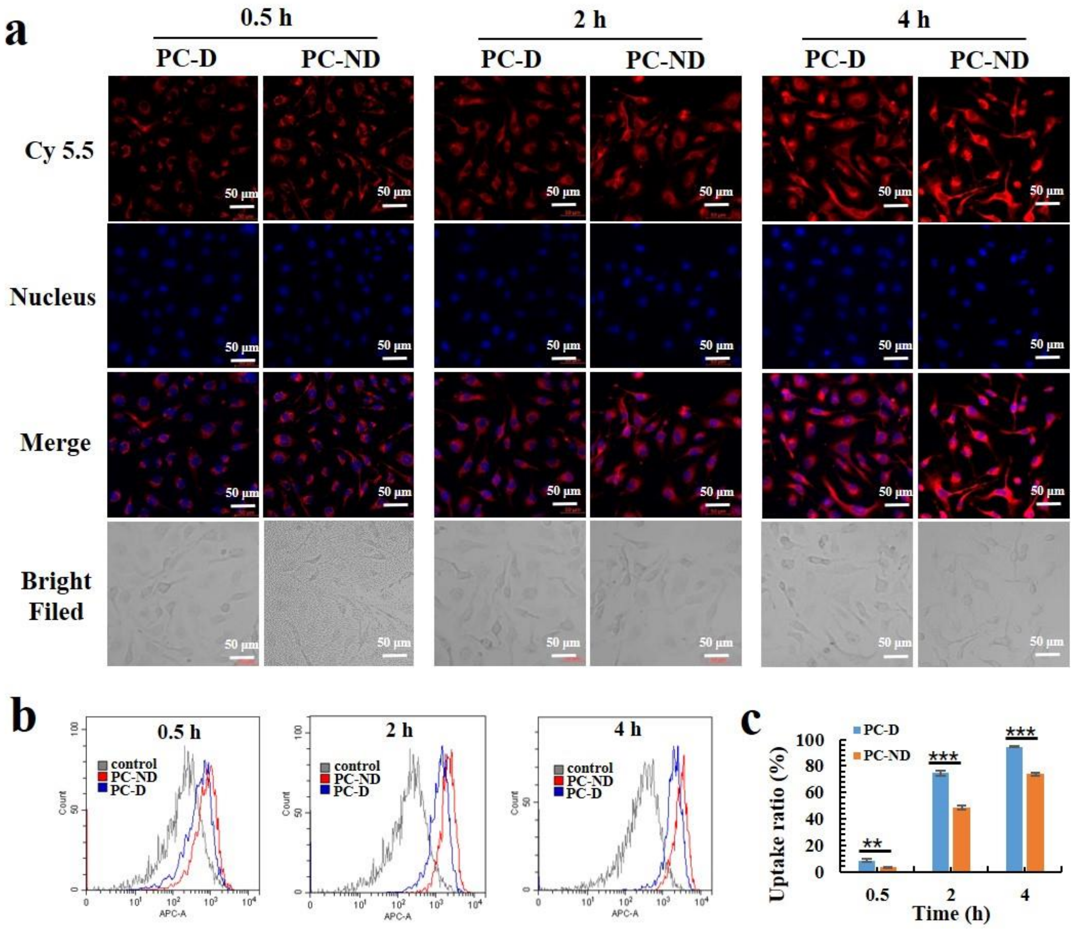

Figure S11. The evaluation of cellular uptake in HUVEC. (a) Laser confocal microscopy (LSM) and (b-c) flow cytometry analysis of cellular uptake of cy5.5 loaded PC-D and PC-ND in HUVEC at different pH values in different time (**$p < 0.01$, ***$p<0.001$, scale bar=50 μm).

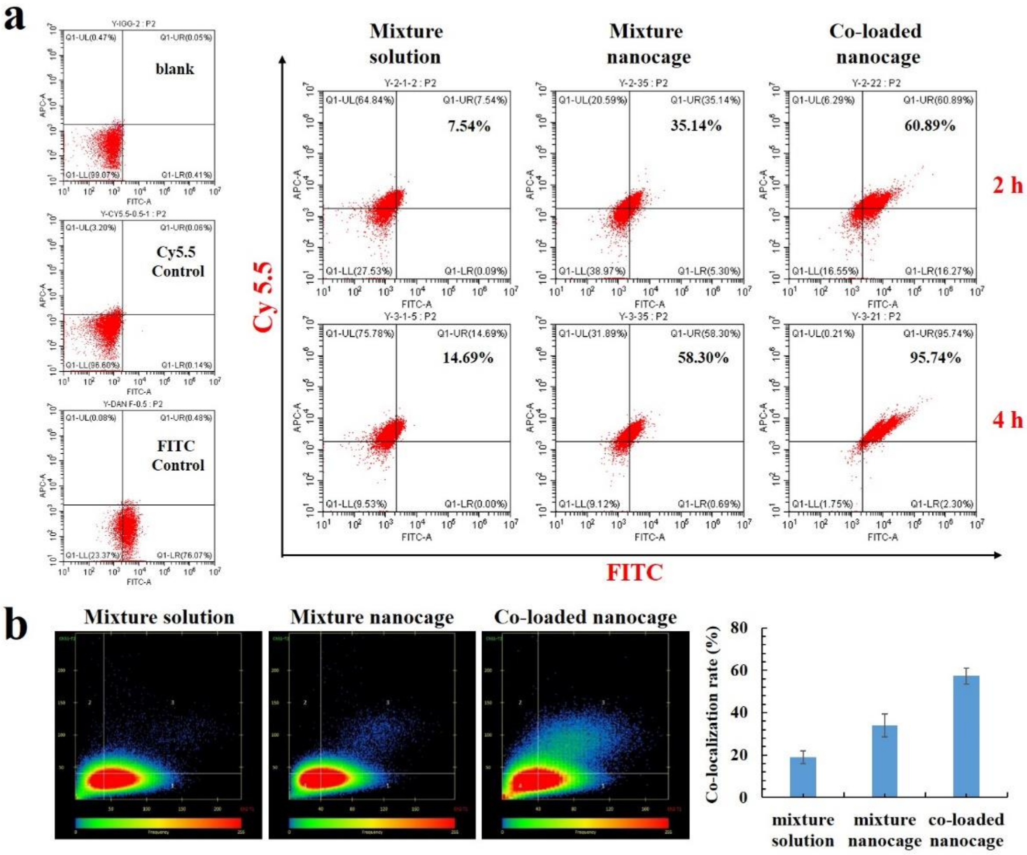

Figure S12. Co-localization efficiency evaluation of PA/PI-ND by (a) flow cytometric analysis in CT26 cells and (b) ZEISS ZEN Lite Software in BALB/c mice.

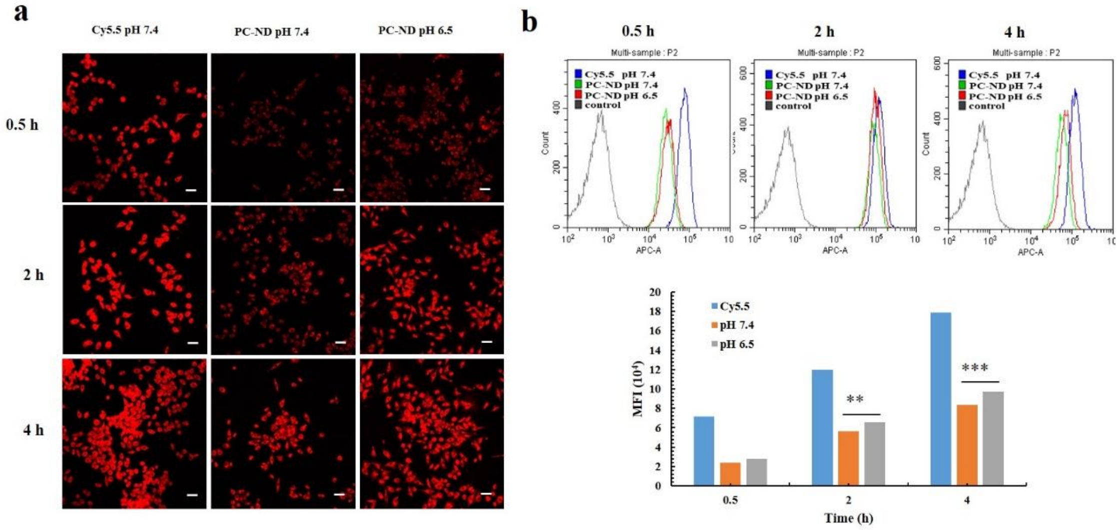

Figure S13. Intracellular uptake evaluation of PA/PI-ND on MCF-7 cells. (a) LSM images and (b) flow cytometric analysis of cellular uptake in MCF-7 cells at different pH values (**p<0.01, ***p <0.001, scale bar=50 μm).

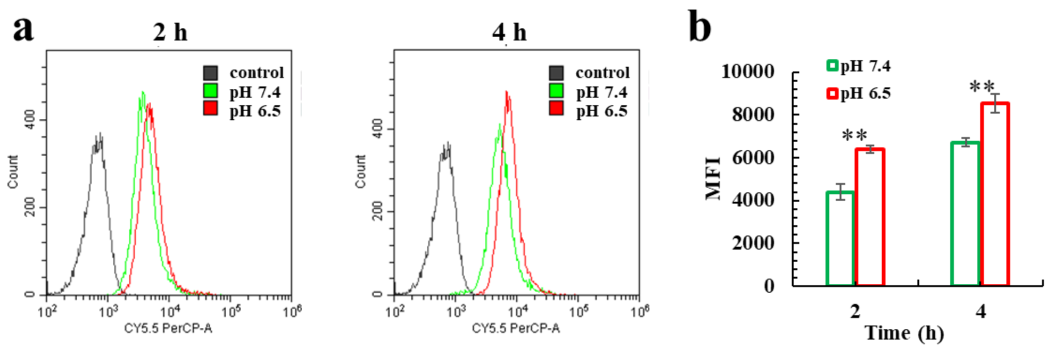

Figure S14. The evaluation of cellular uptake in RAW264.7. (a-b) flow cytometry analysis of cellular uptake of cy5.5 PC-ND in RAW264.7 at different pH values in different time (***p* < 0.01).

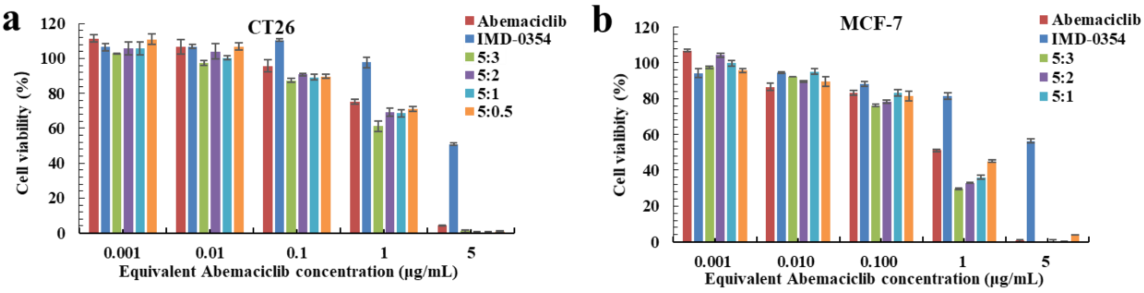

IC_{50} (μg/mL)	Abemaciclib	IMD-0354	5/3	5/2	5/1	5/0.5
$IC_{50(Abemacicilib)}$	1.189	/	0.423	0.560	0.472	0.667
$IC_{50(IMD-0354)}$	/	6.131	0.084	0.112	0.094	0.133
CI	/	/	0.369	0.489	0.412	0.583

IC_{50} (μg/mL)	Abemaciclib	IMD-0354	5/3	5/2	5/1	5/0.5
$IC_{50(Abemacicilib)}$	0.259	/	0.082	0.163	0.139	0.230
$IC_{50(IMD-0354)}$	/	11.21	0.016	0.033	0.028	0.046
CI	/	/	0.317	0.632	0.539	0.892

Figure S15. The evaluation of combination index (*CI*) between abemacicilib and IMD-0354 *in vitro*. Cell viabilities of abemaciclib and IMD-0354 mixtures at different molar ratios in (a) CT26 cells and (b) MCF-7 cells (The concentration was represented abemaciclib, and the concentration of IMD-0354 was accorded with the Abemaciclib/IMD-0354 mass ratios).

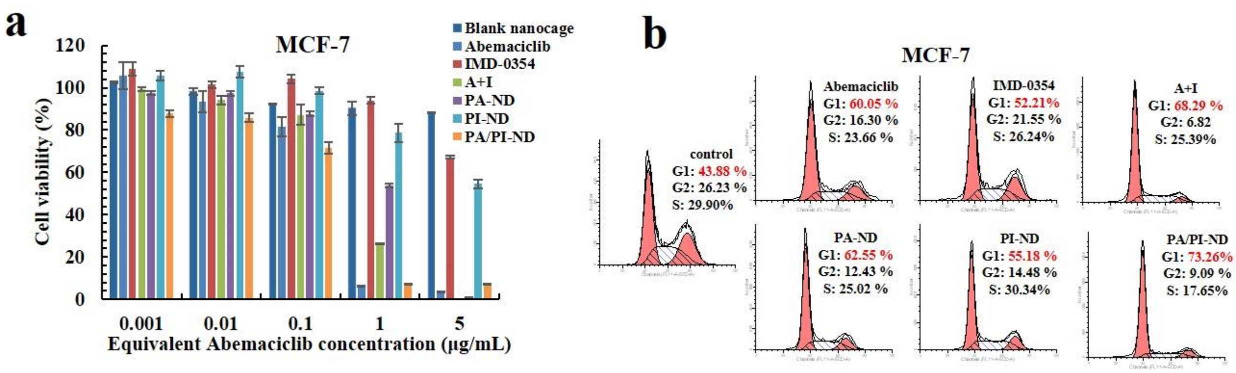

Figure S16. Cytotoxicity and cell cycle arrest of PA/PI-ND *in vitro*. (a) Cell viability of PA/PI-ND and (b) behavior of cell cycle arrest after treated different samples for 24 h on MCF-7 cells *in vitro*.

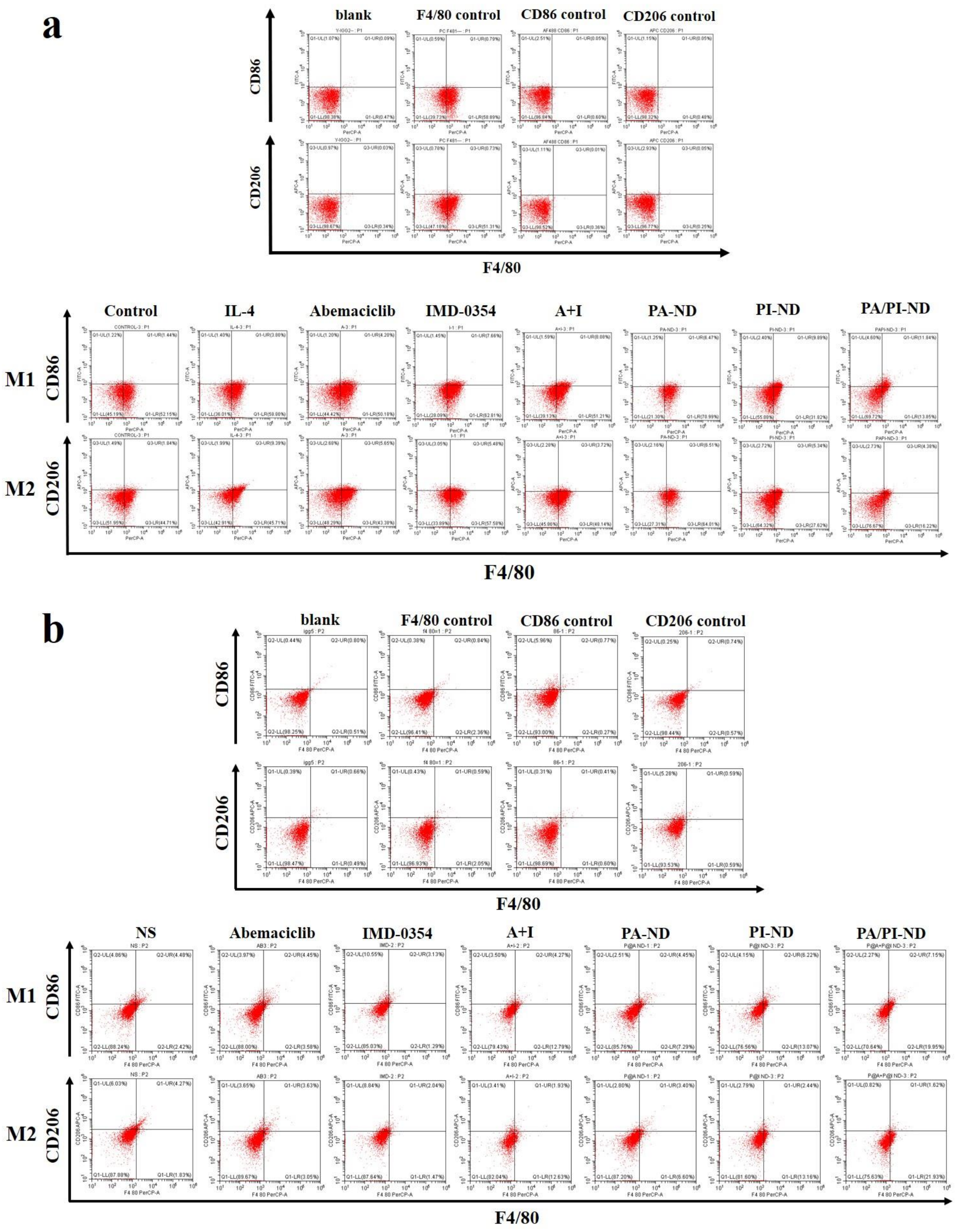

Figure S17. The characterization of TAM phenotype. Flow cytometric analysis of the TAM phenotype (a) *in vitro* and (b) *in vivo*.

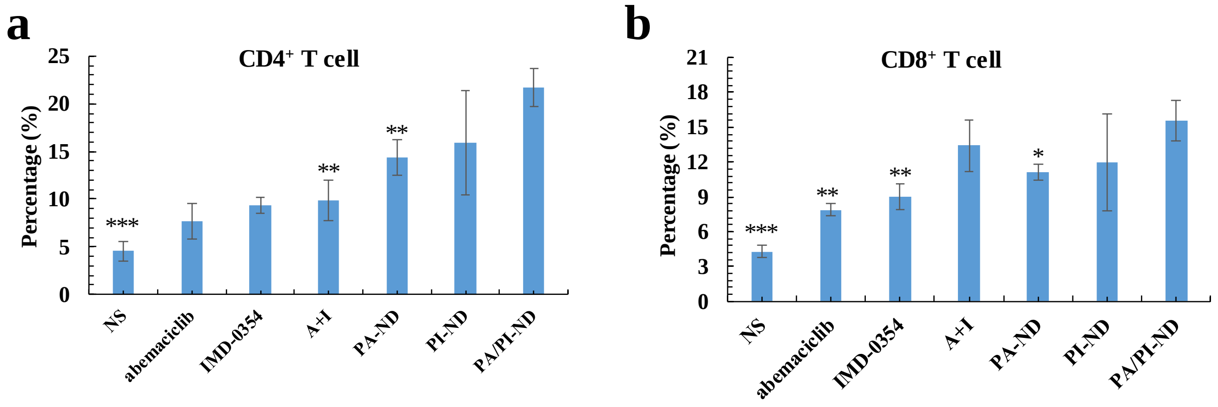

Figure S18. The percentages of (a) CD4+ T cells and (b) CD8+ T cells *in vivo* (*p<0.05, **p<0.01 and ***p<0.001, compared with PA/PI-ND group).

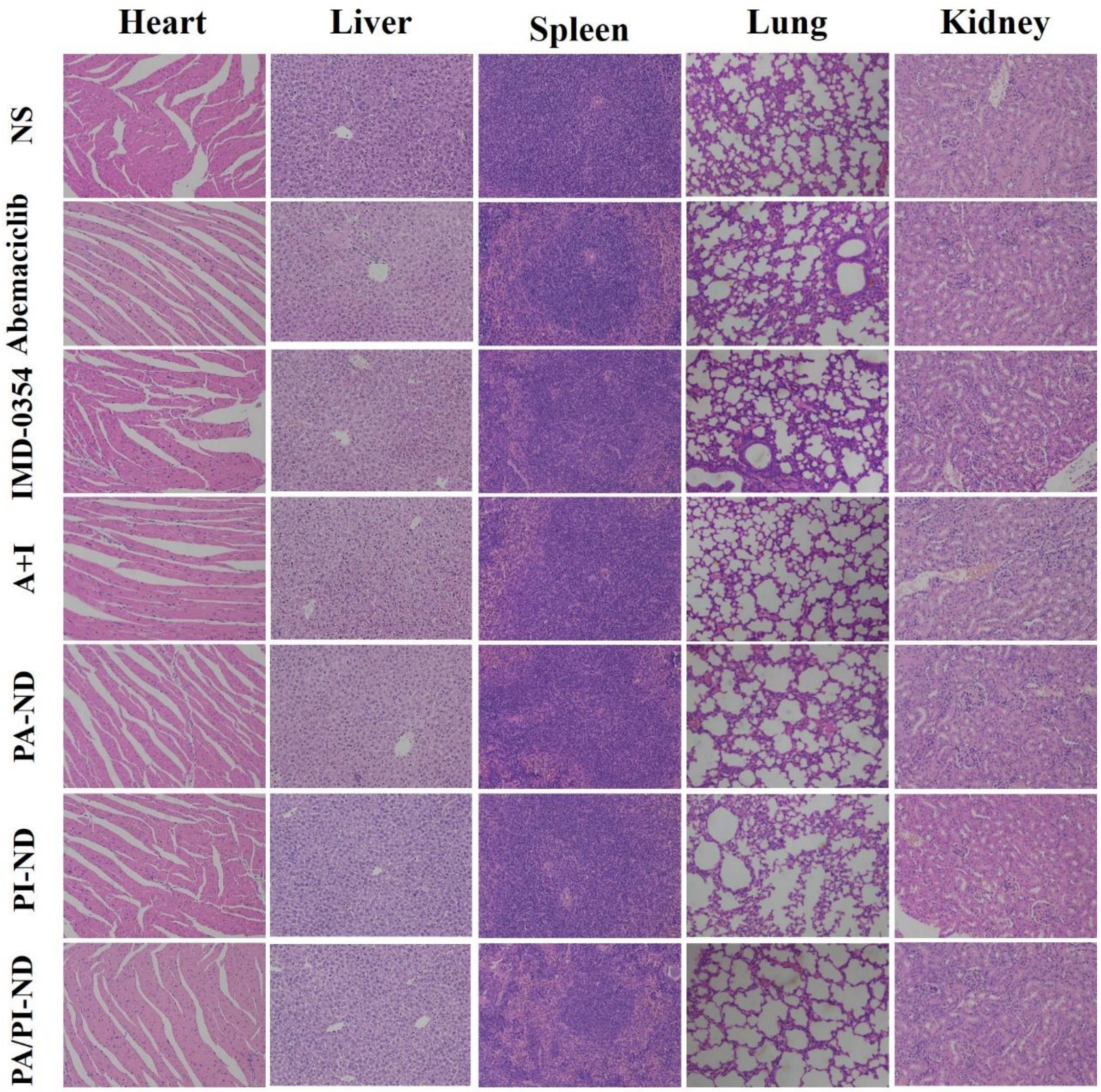

Figure S19. Preliminary safety assessment. H&E stained images of histological sections on CT26

bearing BALB/c mice after treatment with different formulations (scale bar=200×).

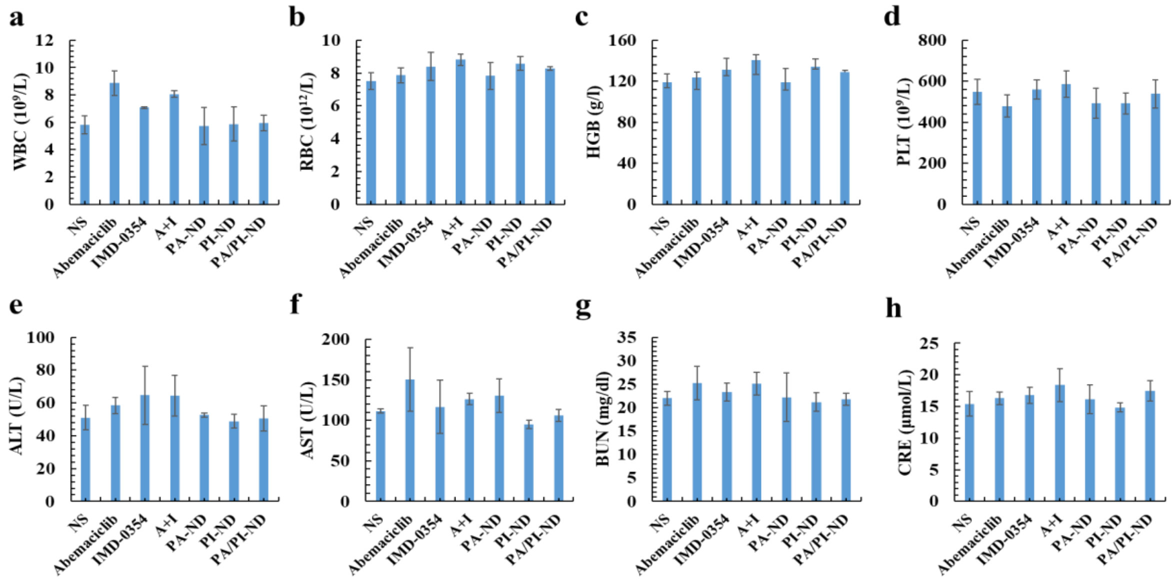

Figure S20. The hematological analysis of mice after treatment different formulations. (a) white blood cell, (b) red blood cell, (c) hematocrit, (d) platelets, (e) alanine aminotransferase, (f) aspartate aminotransferase, (g) blood urea nitrogen and (h) creatinine.

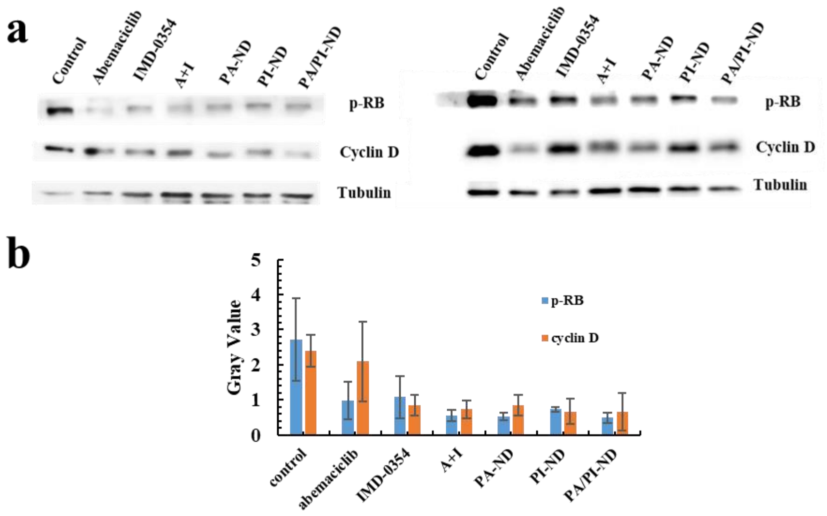

Figure S21. (a) Western blot of CT26 cells after treatment with different formulations for 24 h and (b) the quantitative data of p-RB and cyclin D based on the results of western blot.

Table S1. The GPC of PLL, MAL-PEG-PLL and NGR-PEG-PLL-DMA (ND).

	Mw	PDI
PLL	35575	1.212
MAL-PEG-PLL	137538	1.532
ND	184332	1.732

Table S2. Summary of average sizes, polydispersity indexs (PDI), zeta potentials and drug loadingo f PA, PI and PA/PI-ND.

	Size (nm)	PDI	Zeta (mV)	Abemaciclib DL (%)	IMD-0354 DL (%)
PA	4.59±0.38	0.363±0.05	15.7±0.81	9.38±0.41	---
PI	5.84±1.28	0.495±0.08	15.2±1.16	---	4.33±0.26
PA/PI-ND	177.7±5.807	0.231±0.021	-11.2±0.45	2.79±0.042	1.19±0.006

Table S3. IC_{50} of different samples on (a) CT26 cells and (b) MCF-7 cells after incubated for 48 h, respectively (n=3, **p*<0.05, ***p*<0.01, ****p*<0.001, compared with abemaciclib solution; ##*p*<0.01, compared with PA-ND group)

a

	Abemaciclib	IMD-0354	A+I	PA-ND	PI-ND	PA/PI-ND
$IC_{50\ Abemaciclib}$ （μg mL^{-1}）	0.771±0.095	/	0.307±0.008**	0.742±0.071	/	0.235±0.006***
$IC_{50\ IMD\text{-}0354}$ （μg mL^{-1}）	/	6.131±1.839	0.061±0.002	/	2.857±0.060	0.047±0.001

b

	Abemaciclib	IMD-0354	A+I	PA-ND	PI-ND	PA/PI-ND
$IC_{50\ Abemaciclib}$ （μg mL^{-1}）	0.247±0.058	/	0.113±0.030	0.128±0.018*	/	0.076±0.013**,##
$IC_{50\ IMD\text{-}0354}$ （μg mL^{-1}）	/	5.875±0.437	0.023±0.006	/	0.971±0.790	0.015±0.003
